# Updates and Original Case Studies Focused on the NMR-Linked Metabolomics Analysis of Human Oral Fluids Part II: Applications to the Diagnosis and Prognostic Monitoring of Oral and Systemic Cancers

**DOI:** 10.3390/metabo12090778

**Published:** 2022-08-24

**Authors:** Martin Grootveld, Benita C. Percival, Georgina Page, Kayleigh Hunwin, Mohammed Bhogadia, Wyman Chan, Mark Edgar

**Affiliations:** 1 Leicester School of Pharmacy, De Montfort University, Leicester LE1 9BH, UK; 2 SmileStudio (UK) Ltd., 1st Floor Wingate House, 93-107 Shaftesbury Avenue, London W1D 8BT, UK

**Keywords:** saliva, metabolomics, ^1^H NMR analysis, ^19^F NMR analysis, NMR-based metabolomics, oral cancers, systemic cancers, diagnosis, prognostic monitoring, chemical pathology, GlycA and GlycB biomarkers

## Abstract

Human saliva offers many advantages over other biofluids regarding its use and value as a bioanalytical medium for the identification and prognostic monitoring of human diseases, mainly because its collection is largely non-invasive, is relatively cheap, and does not require any major clinical supervision, nor supervisory input. Indeed, participants donating this biofluid for such purposes, including the identification, validation and quantification of surrogate biomarkers, may easily self-collect such samples in their homes following the provision of full collection details to them by researchers. In this report, the authors have focused on the applications of metabolomics technologies to the diagnosis and progressive severity monitoring of human cancer conditions, firstly oral cancers (e.g., oral cavity squamous cell carcinoma), and secondly extra-oral (systemic) cancers such as lung, breast and prostate cancers. For each publication reviewed, the authors provide a detailed evaluation and critical appraisal of the experimental design, sample size, ease of sample collection (usually but not exclusively as whole mouth saliva (WMS)), their transport, length of storage and preparation for analysis. Moreover, recommended protocols for the optimisation of NMR pulse sequences for analysis, along with the application of methods and techniques for verifying and resonance assignments and validating the quantification of biomolecules responsible, are critically considered. In view of the authors’ specialisms and research interests, the majority of these investigations were conducted using NMR-based metabolomics techniques. The extension of these studies to determinations of metabolic pathways which have been pathologically disturbed in these diseases is also assessed here and reviewed. Where available, data for the monitoring of patients’ responses to chemotherapeutic treatments, and in one case, radiotherapy, are also evaluated herein. Additionally, a novel case study featured evaluates the molecular nature, levels and diagnostic potential of ^1^H NMR-detectable salivary ‘acute-phase’ glycoprotein carbohydrate side chains, and/or their monomeric saccharide derivatives, as biomarkers for cancer and inflammatory conditions.

## 1. Introduction

In Part I of this series, the authors provided a full review and critique of the value of NMR-linked technologies for chemopathological investigations of human saliva and further oral fluids [1]. This included full considerations of the different classes, sources, and biomolecular composition of human saliva as a biofluid, and also the distinction of selected ‘pools’ of metabolites found therein between host, oral microbiome and perhaps other sources; the value of saliva, most notably as whole mouth saliva (WMS), and supernatants arising from its centrifugation (WMSSs), as an acceptable medium for the tracking of disease biomarkers, most especially but not exclusively for oral diseases; the ease of collection routes and processes for this biofluid, and sensible advice concerning the correct protocols to employ for its donation by recruited participants, particularly the minimum fasting abstention period required in order to circumvent issues arising from interfering xenobiotic resonances in salivary ^1^H NMR profiles. Critical regimens evaluated included the transport, storage and preparation of samples for NMR analysis, the latter featuring protocols for the treatment of samples with salivary metabolite-preserving microbicidal agents; optimal pulse sequences for the acquisition of spectra on WMSSs, along with recommended methods for, and the assignment benefits offered by, a range of two-dimensional (2D) NMR strategies; and post-acquisitional preprocessing of the ^1^H NMR spectral profiles of these samples, particularly the chemical shift ‘bucketing’ or ‘binning’ of ^1^H NMR signals, together with finite methods for the quantification of metabolites, and the normalisation, transformation and scaling of multivariate (MV) datasets arising from these profiles. Also featured was a new factor analysis-based strategy for salivary phenotype analysis, which served to effectively distinguish between oral microbiome- or host-dominant, or for that matter admixed, metabolic profiles of human saliva samples. Moreover, the potential future applications of low-field (LF) compact NMR spectrometers for exploring the health status of dental patients, with oral conditions or otherwise, was reviewed, with special reference to their employment at point-of-contact sites such as dental surgeries or pharmacies. 

Primarily, Section 1 of this report provides a short appraisal of the many advantages offered by ‘state-of-the-art’ high-resolution ^1^H NMR analysis techniques for the metabolomics analysis of human saliva samples. Also covered are typical WMSS ^1^H NMR resonance assignments, along with some major recent improvements in this technique’s selectivity and sensitivity, and its ability to rapidly acquire such data at maximal levels of laboratory efficacy. Although unusual, this section then briefly explores corresponding developments in high-field ^19^F NMR analysis, and its potential relevance to both diagnostic and drug-tracking metabolomics strategies is discussed. A further sub-section devoted to the importance of metabolomics investigations to clinical epidemiology is also included here. Subsequently, essential information focused on the metabolic pathways which facilitate the sustenance of cancer cell longevity and proliferation is provided (Section 2), along with a brief overview based on the applications of NMR-linked metabolomics analysis for the screening of saliva samples for differential classes of cancers in general, including recently conducted systematic reviews (Section 3). Subsequently, Section 4 begins with the application of these approaches towards the diagnosis and prognostic monitoring of a series of oral cancers (OCs) in humans (notably potentially malignant oral condition (PMOC) prequelae, and oral cavity squamous cell carcinoma (OCC) and oropharyngeal squamous cell carcinoma (OPC)), whereas Section 5 features the use of human saliva for the detection and monitoring of a wide range of extra-oral (systemic) cancers, notably head and neck, squamous cell, lung, breast, pancreatic and prostate cancers, amongst others. Following this, Section 6 provides a review of information provided in a recent case study based on a common adverse response to radiation therapy applied in the treatment of head and neck cancer (HNC), specifically oral mucositis (OM). Finally, in view of their now recognised and increased applications to explore and determine the pathological status of a wide range of cancers and inflammatory conditions, Section 7 features a new case study involving the very first evaluation of the ^1^H NMR signals of ‘acute-phase’ glycoproteins detectable in WMSS samples, groundwork which was conducted for the first time here in order to potentially establish their value as diagnostic biomarkers in cancers, in addition to other diseases, notably inflammatory ones. Also explored are the potentially confounding roles of interferences in the ^1^H NMR determination of such critically important biomacromolecules. Additionally, Section 8 discusses the clinical implications of salivary metabolomics studies. Section 9 then provides full details of the reliable employment of such ‘big’ metabolomics datasets to inform researchers of dysregulated or imbalanced metabolic pathways so that drug targets may be identified, developments which may then in turn lead to drug discovery programmes. This section also includes the potential use of these multianalyte metabolomics strategies to inform clinicians on suitable drug treatment options for cancers. Finally, limitations of the applications of NMR-based salivary metabolomics techniques to evaluate key biomarkers for the potential diagnosis and tracking of human cancers are provided in Section 10, and this is followed by a series of generalised conclusions for this study (Section 11).

Of key importance, novel supporting disease screening protocols, facilities and devices are urgently required in order to combat morbidity and mortality in cancer conditions, and therefore heightened efforts should be made by those involved in salivary metabolomics investigations of human saliva, in order to further facilitate and/or verify early diagnoses. 

### 1.1. Appraisal of Benefits Offered by the ^1^H NMR Analysis of Human Saliva 

**Figure 1:**
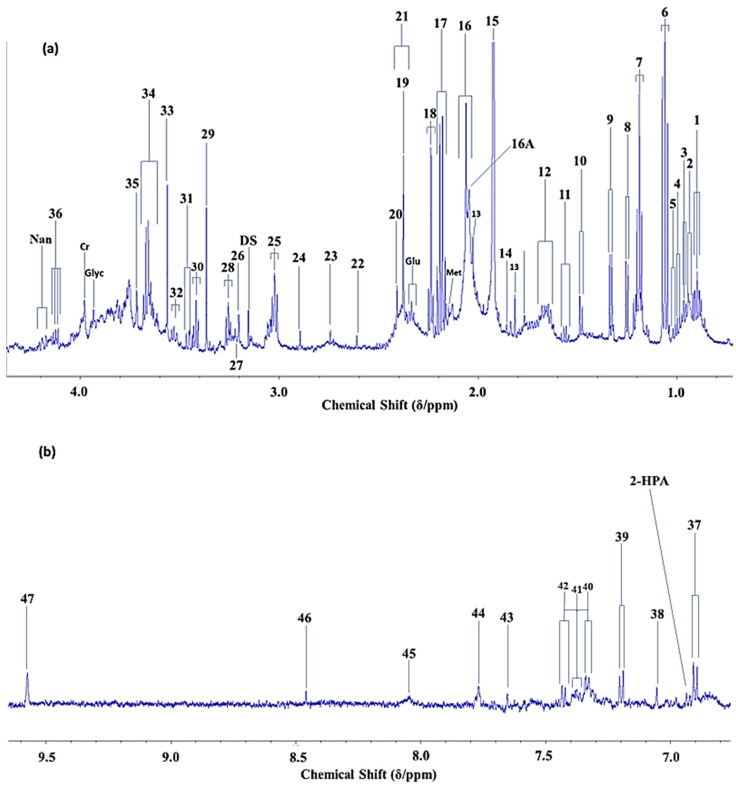

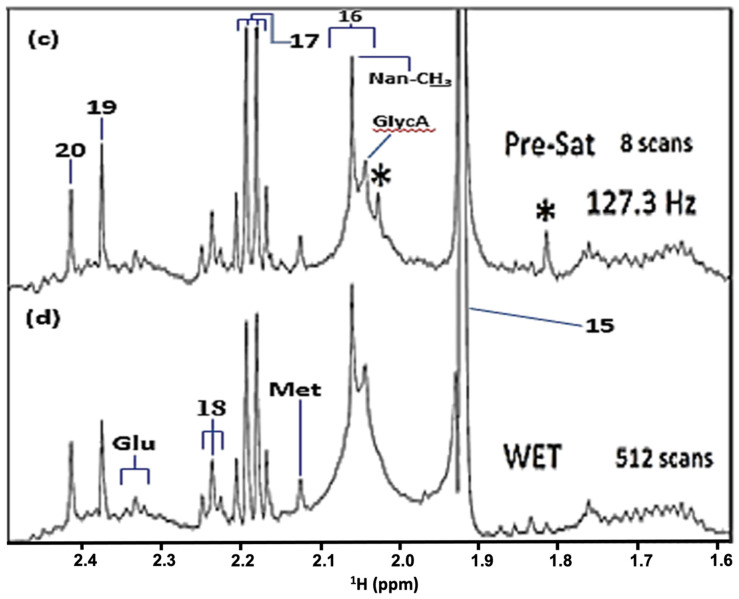
High-Resolution ^1^H NMR Analysis of Human Salivary Supernatant Samples. (**a**,**b**), High- and low-field regions (0.70–4.40 and 6.75–9.65 ppm, respectively) of the 600 MHz *noesy-presat* ^1^H NMR spectrum of a WMSS sample donated by a healthy human participant. Samples were prepared according to the methods described in Refs. [1,2], and contained added phosphate buffer to control pH, azide as a microbicide to curtail metabolite consumption/fermentation and catabolite generation by bacteria during periods of sample preparation and storage prior to analysis, and 250 μmol./L TSP as a chemical shift reference (δ = 0.00 ppm) and quantitative ^1^H NMR standard. (**c**,**d**), 1.60–2.50 ppm regions of repeat *noesy-presat* and WET pulse sequence ^1^H NMR profiles acquired on a human WMSS sample for 512 and only 8 scans, respectively. The *noesy-presat* or WET pulse sequences were employed to suppress the intense water resonance. Typical spectra are shown. Resonance assignment number codes and abbreviations are listed in Table 1. The asterisks in (**c**,**d**) represent ^13^C satellites of the very intense WMSS acetate-CH_3_ resonance.

Figure 1a,b show the expanded high- and low-field regions, respectively, of a 600 MHz *noesy-presat* ^1^H NMR spectrum of a typical whole-mouth salivary supernatant (WMSS) sample collected from a healthy human participant. In addition to a wide range of resonances arising from bacterial organic anion catabolites such as acetate, lactate, fumarate, propionate, *n*-butyrate, succinate and formate, etc. (biomolecules predominantly, but not exclusively, arising from the salivary microbiome), these spectra contain many further signals, e.g., amino acids, carbohydrates including N-acetylsugars, purines and pyrimidines, and lipids etc. Also shown are the results of experiments which were set up to investigate spectral quality as a function of the number of ^1^H NMR scans performed on a single WMSS sample, in this case using both the *noesy-presat* and WET pulse sequences. Clearly, the spectral quality achieved on our 600 MHz spectrometer was very good, and only small differences between such profiles obtained from the use of only 8 scans, and those acquired using as many as 512 scans were discernible. However, determinations of the signal-to-noise (STN) ratio for the TSP quantitative internal standard and chemical shift reference (δ = 0.00 ppm) revealed an improved sensitivity for the 512-scan profile in *noesy-presat* spectra acquired, i.e., STN values of 267 versus 1241 for 8 and 512 scans, respectively, i.e., a nearly five-fold enhancement for the latter. However, the achievement of high quality biofluid spectra with only 8 scans here is particularly notable, and this has not been previously reported on WMSS samples.

**Table 1:** ^1^H NMR Resonance Assignments for Human WMSS Samples at an Operating Frequency of 600 MHz. * Broad resonance located at δ = 8.05 ppm, which along with those at ö = 6.85 and 7.55 ppm [1] (also visible in Figure 1b), presumably arise from salivary protein tyrosine, phenylalanine, tryptophan and/or histidine residues. ** This aldehydic proton (-CHO function) signal may be a singlet or a triplet; if a triplet, as in saturated aldehydes (*n*-alkanals), without expansion it appears as a singlet in view of a very low *J* coupling constant value (1.4 Hz) [3]. Abbreviations: APP, acute-phase protein; *s*, *d*, *t*, *q* and *m*, singlet, doublet, triplet, quartet and multiplet resonance multiplicities, respectively.

Assignment Number/Code	Chemical Shift (δ/ppm)	Multiplicity	Assignment
1	0.92	*t*	*n*-Butyrate-CH_3_
2	0.94	*broad*	Protein BCAA side-chain-CH_3_
3	0.96	*t*	Leucine-CH_3_
4	0.97	*d*	Valine-CH_3_
5	1.02	d	Valine-CH_3_
6	1.06	*t*	Propioniate-CH_3_
7	1.20	*t*	Ethanol-CH_3_
8	1.13	*d*	*iso*-Butyrate-CH_3_
9	1.33	*d*	Lactate-CH_3_
10	1.48	*d*	Alanine-CH_3_
11	1.57	*tq*	*n*-Butyrate-β-CH_2_
12	1.65	*m*	5-Aminovalerate-CH_2′_s
13	1.80, 2.028	2 x *s*	Acetate-CH_3_^13^C satellites
14	1.87	*s*	Thymine-CH_3_
15	1.92	*s*	Acetate-CH_3_
16	1.95–2.10	*broad/sharp s*	Broad: Glycoprotein -NHCOCH_3_/Sharp: Free Aminosugar- and N-Acetyl-amino acid-NHCOCH_3_
GlycA	2.040	*s*	GlycA APP N-Acetylglucosamine residues
Nan-CH_3_	2.06	*s*	Free N-Acetylneuraminate
Met	2.13	*s*	Methionine-S(CH_3_)_3_
17	2.17	*q*	Propioniate-CH_2_/*n*-Butyrate-α-CH_2_
18	2.23	*t*	5-Aminovalerate-CH_2_-CO_2_^−^
Glu	2.36	*m*	Glutamate--β-CH_2_
19	2.38	*s*	Pyruvate-CH_3_
20	2.405	*s*	Succinate-CH_2′_s
21	2.39	*m*	Isobutyrate-CH
22	2.59	*s*	Methylamine H_2_NCH_3_
23	2.75	*s/m*	DimethylamineH_2_N(CH_3_) /Methionine-CH_2_
24	2.95	*s*	Trimethylamine N(CH_3_)_3_
25	3.04	*t*	5-Aminovalerate-5-CH_2_/Lysine-ε-CH_2_
DS	3.145	*s*	Dimethylsulphone-OS(CH_3_)_2_
26	3.21	*s*	Choline-N(CH_3_)_3_^+^
27	3.24	*s*	Betaine-N(CH_3_)_3_
28	3.25	*t*	Taurine-CH_2_NH_3_^+^
29	3.38	*s*	Methanol-CH_3_
30	3.43	*t*	Taurine-CH_2_SO_3_^−^
31	3.46	*d*	*cis*-Aconitate-CH_2_
32	3.54	*dd*	Glycerol-CH_2_OH
33	3.56	*s*	Glycine-CH_2_
34	3.66	*q/m*	Ethanol-CH_2_/Glutamate-α-CH
35	3.72	*m*	Leucine-α-CH
Glyc	3.92	*s*	Glycolate-CH_2_
Cr	3.95	*s*	Creatine-N(CH_3_)
Nan	4.02	*m*	N-Acetylneuraminate-C4H
36	4.13	*q*	Lactate-CH
37	6.88	*d*	Tyrosine-Aromatic ring protons
2-HPA	6.93	*m*	2-Hydroxyphenylacetate-Aromatic ring proton
38	7.06	*s*	Histidine-Imidazole ring protons
39	7.20	*d*	Tyrosine-Aromatic ring protons
40	7.32	*m*	Phenylalanine-Aromatic ring proton
41	7.36	*m*	Phenylalanine-Aromatic ring proton
42	7.42	*m*	Phenylalanine-Aromatic ring proton
43	7.65	*s*	Guanine-CH=
44	7.78	*s*	Histidine-Imidazole ring protons
45	8.05	*broad*	* Protein aromatic amino acid residue(s)
46	8.45	*s*	Formate-CH
47	9.57	** s(t)	Unassigned saturated aldehyde-CHO function

### 1.2. ^19^F NMR Analysis of Human Saliva, Oral Biopsies and Tap Water 

**Figure 2:**
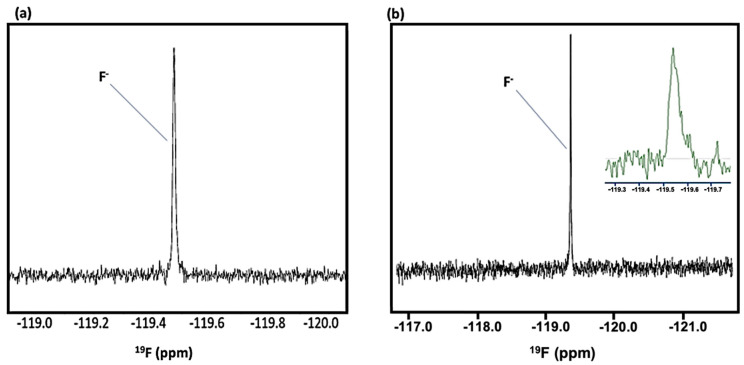
Newly Developed Biomedical Applications of High-Field ^19^F NMR Spectroscopy: Analysis of Human Saliva and Tap Water. ^19^F NMR spectra of (**a**) an aqueous sodium fluoride calibration standard (δ*_F_* = −119.5 ppm; final analyte concentration 17.6 µmol./L) and (**b**) a WMSS sample collected from a participant following an 8.00 h. oral abstention ‘fasting’ period. Analysis solutions contained 527 µL of a 20 µmol./L sodium fluoride solution, 60 µL of ^2^H_2_O and 13 µL of a 50.00 mmol./L trifluoroacetate (TFA) internal standard (δ*_F_* = −75.3 ppm). ^19^F NMR spectra were acquired on a JNM-ECZ600R/S1 600 MHz NMR spectrometer (operating at a frequency of 564.72 MHz for ^19^F), over a 400 ppm spectral width and with an FID acquisition time of 0.92 s. An 8.3 µs 90° pulse was used; the relaxation delay between pulses was 3 s. In total, 2048 scans were acquired with 256 K data points, which were then Fourier-transformed with zero-filling to 512 K data points and a single exponential function of 5.0 Hz. Baseline roll signal from the fast-relaxing fluoropolymer species in the NMR probe-head was removed using backward linear prediction (order = 16, sample data 512 points, reconstructed data 32 points); baseline correction was applied to spectra using a polynomial function. The resulting signal-to-noise (STN) ratio for the ^19^F resonance in (**a**) was 50:1. Chemical shift values were referenced to external fluorotrichloromethane (CFCl_3_; Jeol UK Ltd. default reference setting). **Inset:** Partial ^19^F NMR spectrum of a local, East Midlands, UK, sample of tap water (shown in green) demonstrating the detection of fluoride anion therein; this spectrum was also acquired with backward linear prediction and 2048 scans.

Of more general interest, and in support of the wide range of multinuclear advantages and biomedical applications of high-resolution NMR spectroscopy, together with its ability to identify and quantify many biomolecular and/or xenobiotic analytes simultaneously and rapidly, Figure 2 shows the ^19^F NMR spectra of a typical WMSS sample, and also a very low concentration fluoride calibration standard solution, the former clearly demonstrating the direct detection of traces of fluoride in this biofluid. For this purpose, we employed a trifluoroacetate (TFA) internal standard (δ = −75.3 ppm). The fluoride anion concentration of the above standard was 20 µmol./L (0.38 ppm), a value which is very similar to its mean baseline human salivary level of 0.41 ± 0.38 ppm (mean ± SD) [4] (equivalent to a mean value of 21.6 µmol./L), although it should be noted that fluoride was undetectable in at least some baseline control WMSS samples explored with this technique; participants fasted for an 8 h. duration prior to providing samples. 

The major barrier for this analysis was overcoming the interfering very broad, fast-relaxing fluorine resonances which arose from a solid fluoropolymer present in the NMR magnet probe-head. However, this was effectively achieved by the removal of data points from the start of the FID which contributed to these very broad signals in the spectrum, followed by the use of part of the FID in order to predict and replace that which was removed. This process resulted in a flatter baseline, with only the much narrower F^−^ and TFA resonances remaining unchanged. Importantly, this processing of the FID is mathematically robust and reproducible. 

These spectra clearly demonstrate that, for the first time, ^19^F NMR analysis can be readily employed to monitor F^−^ levels in human saliva, and also perhaps to ‘speciate’ fluorine in this biofluid. There are also clear applications of this strategy to the analysis of fluorine-containing agents such as dentifrice fluorophosphate and fluoro-substituted drugs, along with fluoride itself, in oral biopsies, for example, in primary root carious lesions. Also shown is the very first direct ^19^F NMR detection and quantification of tap water fluoride in a UK (East Midlands) city, a non-artificially fluoridated area—according to information available in Ref. [5], this level should lie somewhere within the 0.10–0.70 ppm (5–37 µmol./L) range. The STN parameter determined for the above 20.0 µmol./L (final analyte solution level 17.6 µmol./L) fluoride calibration standard was 50, so therefore levels as low as 3–4 µmol./L are quantifiable in biofluids and environmental samples with a corresponding STN value of ca. 10. Clearly, these STN values and quantification limits will be enhanced and diminished somewhat, respectively, by markedly increasing the number of NMR spectral scans made over and above our value here of 2048 during acquisition. Quantitative ^19^F NMR analysis of this calibration standard solution yielded an acceptable estimate of its fluoride concentration of 21.4 µmol./L, i.e., a value within 7% of its known level. These pioneering ^19^F NMR analysis studies will be reported in more detail elsewhere. 

Intriguingly, ^19^F NMR analysis may also be employed for investigating the mechanisms of action and dispositions of fluorine-containing anti-cancer agents, and the development and testing of fluorine-containing chemotherapeutic agents (such as 5-fluorouracil), which can act as powerful molecular ‘warheads’ in cancer treatment when linked to appropriate tumour-targeted drug delivery systems [6]. Further developments have included the design and synthesis of fluorine-containing toxoids, i.e., fluorotaxoids [6]. Hence, ^19^F NMR analysis is likely to offer valuable molecular information regarding the therapeutic monitoring of these novel therapeutic agents, their prodrugs and metabolites in biofluids or tissue biopsies collected from selected groups of human patients, including those with deficient catabolising enzymes for fluorouracil, or those undergoing haemodialysis. In addition to having much relevance to salivary diagnostics for cancer conditions, these ^19^F NMR investigations will be valuable for probing the potential therapeutic activities of fluorocarbon drugs, and for monitoring their biodistributions and effective targeting of tumours and associated tissues. 

### 1.3. Importance of Metabolomics Investigations in Clinical Epidemiology

Currently, metabolomics techniques are becoming increasingly important and common in clinical epidemiology in view of the advent of newly developed quantitative profiling and sensitivity advantages of the technologies applied (e.g., ^1^H NMR spectroscopy, LC-MS, etc.), and highly valuable metabolite datasets which arise from their implementation facilitate our understanding of the biomolecular basis of human health and disease states. Indeed, the majority of clinical epidemiological investigations frequently involve the determination of a panel of blood plasma or serum sample biomarkers, for example, glucose, lipoprotein cholesterol, creatinine, total protein, etc., for probing the health and disease status of human populations. Since high-resolution ^1^H NMR analysis represents a high-throughput technique which may simultaneously identify and quantitate very large numbers of metabolites in a single biofluid (e.g., >120 or so in human urine) within a short period of time, this platform is readily applicable to such clinical epidemiological investigations featuring very large numbers of participants, although the correct standardisation of protocols for sample collection and laboratory preparation methods is an essential pre-requirement, as are many other factors. Such studies also require rigorous experimental design and modelling considerations prior to proceeding, so that all possible contributory ‘input’ variables are incorporated, including major demographic variables such as age, gender and body mass index (BMI), together with known or perceived risk factors, etc., in order to optimise the value and precision of data acquired, hypothesis-driven or otherwise. Examples of the use of the ^1^H NMR-based metabolomics technique to large-scale clinical epidemiological studies include its use for the detection and measurement of biomarkers for early atherosclerosis [7], type 2 diabetes mellitus [8], diabetic nephropathy [9], coronary heart disease [10] and all-cause mortality [11].

The research group that undertook these investigations have also taken various routes towards multi-omics systems epidemiology, for example, to understand liver function [12] and to identify causal networks of gene expression modules [13]. Interestingly, these researchers state the total cost of a single ^1^H NMR screen of blood plasma or serum, featuring the provision of finite bioanalytical data on a range of cholesterol marker indices, a plethora of lipoprotein classes, many low-molecular–mass biomolecules such as proteinogenic and some non-proteinogenic amino acids, glycolysis pathway-associated metabolites, diabetes-relevant ketone bodies, creatinine and carbohydrates, the latter including glucose and acute-phase protein (APP)-linked N-acetylsugars as residues present in their molecularly-mobile carbohydrate side-chains. According to these reports, all of these metabolite and biomarker determinations are acquirable from a single blood plasma or serum sample at economic costings which are comparable with those of standard lipid assays for clinical monitoring purposes. 

Similar powerful arguments are also likely to apply to the use of human saliva for such purposes, which offers additional benefits, including ease of sample collection, handling and analytical preparation, along with its ability to detect and predominantly quantify 100 or so metabolites simultaneously at NMR operating frequencies of 600 MHz or higher [1]. However, to the best of our knowledge, to date there appears to be little or no application of salivary NMR-based metabolomics in addressing the critical sample analysis requirement within large-scale clinical epidemiological cohort studies. Hence, there remains a major exigency for the wide-scale, perhaps global use of these technologies in such typically widespread or global investigations, most especially because of the lowered costs associated with the collection and handling of this biofluid when comparatively evaluated against those for blood plasma or serum analysis. 

Hence, the ^1^H NMR-based metabolomics analysis of human saliva samples has much to offer in the context of large-scale clinical epidemiological studies, and after making allowances for selected restrictions, could be factored in as a major work task for such studies, not only for oral diseases including cancers, but also systemic ones which may already employ validated salivary biomarkers reliably for diagnosis and monitoring purposes. 

For such studies, the increasing build-up of valuable quantitative ^1^H NMR data based on systemic metabolism is continually evolving, and for the last 20–30 years or so has now demonstrated a multitude of new biomarkers. Indeed, in our laboratory, it is only on very rare occasions that our researchers fail to detect one or more new or novel, previously undiscovered molecular species when conducting just about any NMR-linked metabolomics experiment. This confirms that quantitative NMR (qNMR) and associated MV metabolomics or computational intelligence analyses will inevitably transmute and remodel the practices of both clinical epidemiology and genetics. 

## 2. Metabolic Pathways That Sustain Cancer Cell Survival and Proliferation

To date, much has been learnt on the major metabolic pathways dysregulated in cancer conditions, and this information has allowed the development of drug-targeting strategies, and the design and testing of targeted drugs [14]. In general, both catabolic and anabolic routes are upregulated by cancer cells in order to optimise energy and biomacromolecule generation. Indeed, glucose and glutamine represent key biomolecules which have the ability to furnish cancer cells with the majority of energy which is essential for their growth and proliferation, and also serve as metabolite sources for their evolution. Primarily, glucose is taken up by tumour cells via the glucose transporter 1 (GLUT1) transporter system and then enters the glycolysis pathway, and the glucose-6-phosphate glycolytic intermediate may then be diverter-routed to the pentose phosphate pathway, generating ribose-5-phosphate (nucleotide synthesis), along with electron-donating equivalents of NADPH (anabolic activities). However, a further glycolytic intermediate, 3-phosphoglycerate, can be redirected to the biosynthesis of both glycine and serine, which can then be integrated into protein or nucleotide structures, or employed as sources of other metabolites. Lastly, pyruvate arising from the glycolysis pathway can be reduced to lactate by lactate dehydrogenase, oxidised in the mitochondrial tricarboxylic acid (TCA) cycle, or transformed to citrate, which serves as a precursor for the biosynthesis of cholesterol and fatty acids (FAs) [7]. 

HMG-CoA reductase acts as the pivotal enzyme for cholesterol biosynthesis, whereas FA biosynthesis is dependent on the availability of acetyl-CoA carboxylase (ACC) and FASN enzymes. The SLC1A5 amino acid transporter delivers glutamine to tumour cells for the purpose of protein or nucleotide biosynthesis; however, it can also be metabolised to glutamate, and subsequently α-ketoglutarate, which may then either be oxidatively converted within the mitochondrial TCA cycle, or reductively metabolised to citrate, and in this context also contributes towards FA and cholesterol biosynthesis. Moreover, transamination of cytoplasmic glutamine generates further amino acids from their corresponding α-ketoacid anions. Specific transporters are employed for the uptake of both methionine and arginine from external locations, which are then utilised for protein biosynthesis or alternative functions [14]. 

Metabolism in cancer cells is highly complex, and potentially displays much heterogeneity within large tumour masses. Fortunately, recent technical developments, which include in vivo magnetic resonance spectroscopy (MRS), along with hyperpolarised magnetic resonance imaging (MRI), may serve to provide a rather detailed molecular ‘picture’ of modifications in metabolite usage and generation in human tumours in vivo, notably at differential disease sites. Such approaches may also be valuable for the prognostic monitoring of the dynamic progression of tumour metabolism during disease evolution, or during their responses to chemotherapeutic treatments administered [15,16]. Indeed, we look forward to the future applications and developments of these techniques in the oral cancer areas. 

Briefly, an upregulated level of aerobic glycolysis offers specific advantages towards cancer cell growth and proliferation [17]. Primarily, when present at blood physiological levels, glucose engenders an acceptably rapid rate of ATP synthesis in order to satisfy energetic requirements, and concomitantly empowers anabolic pathways via the generation of biomass. Moreover, lactate arising from the reduction of pyruvate gives rise to an acidic extracellular environment that enables the recruitment of immune cells such as macrophages, a process facilitating metastasis. Finally, pyruvate can be converted to oxaloacetate, together with alanine and aspartate, which of course are involved in the biosynthesis of proteins or further metabolites [17,18,19].

## 3. Overview of the Metabolomics Screening of Saliva Specimens for Differential Groups of Cancer Conditions, including Selected Systematic Reviews Conducted: Applications to Diagnosis, Prognostic Severity Monitoring and Metabolic Pathway Dysregulations 

This evaluation will be commenced with a very recent systematic review conducted by Assad et al. in 2020 [20], which was focused on an assessment of salivary metabolites as valuable diagnostic biomarkers in cancer patients. This review was constructed and performed in two stages, and also investigations featuring determinations of the diagnostic potential of salivary biomolecules in cases of solid malignant neoplasms. Overall, five electronic databases were searched, and the revised Quality Assessment of Diagnostic Accuracy Studies criteria (QUADAS-2) was employed to compute the risk of bias. Moreover, all criteria were conducted according to Preferred Reporting Items for Systematic Reviews and Meta-Analyses (PRISMA) guidelines. A grand total of 1151 studies were reviewed, and of these only 25 were selected for further evaluation: 13 targeted, and 12 untargeted metabolomics studies, the majority focused on oral and breast cancers. Of 140 salivary biomarkers found, the most popular were alanine, leucine and valine. Moreover, of the 11 investigations which reported diagnostic test accuracy (DTA) parameters, proline, histidine, threonine and monoacylglycerol(s) had the greatest values for breast cancer. Furthermore, a combination of betaine, choline, L-carnitine and pipecolinate had the highest distinctive potential for the early stages of oral cancer conditions. Notwithstanding, as part of a minor case study, the authors of the current investigation performed qualitative pathway topological analyses featuring these ‘validated’ dysregulated metabolite biomarkers available for firstly breast, and secondly oral cancers. However, this strategy failed to detect any significant perturbations to human metabolic pathways in both cases considered, an observation presumably reflecting the low numbers of viable biomarkers detectable in the report documented in [20]. 

From Ref. [20], it was concluded that additional investigations with larger sample sizes are required, along with those featuring the validation and confirmation of results acquired from untargeted analysis. Notwithstanding, all investigations reported in this particular systematic review had case-control designs, although none of them completely satisfied all quality assessments made. 

Additionally, for the primary purpose of the current study, we also describe an extensive investigation conducted by Sugimoto et al. [21] reported in 2010, who performed a fully comprehensive metabolomics analysis of saliva samples donated by patients with oral (n = 69), pancreatic (n = 18) and breast cancers (n = 30), along with 11 periodontal (PD) patients and 87 healthy controls. For this study, capillary electrophoresis time-of-flight mass spectrometry (CE-TOF-MS) was employed as a metabolic screening tool. Overall, a total of 57 lead biomolecules with the ability to accurately predict the probability of being afflicted by each of the above diseases were discovered. However, despite being statistically significant, only low correlations were found between patient disease characteristics and biomarkers determined. Nevertheless, the metabolic profiles displayed relatively higher levels of the majority of the biomarkers identified in all three cancers when compared to those of PD and healthy control participants, and this observation indicated that cancer-specific patterns were enrooted within salivary metabolite profiles. Indeed, three individual ‘pools’ of salivary biomarkers found to be valuable for distinguishing between oral cancer patients and healthy controls consisted of (1) pyrroline hydroxycarboxylate, leucine/isoleucine, choline, tryptophan, valine, threonine, histidine, pipecolate, glutamate, carnitine, alanine, piperidine and taurine, plus two further metabolites; (2) piperidine, α-aminobutyrate, phenylalanine and another (unidentified) metabolite; and (3) betaine, serine, tyrosine, glutamine, β-alanine and cadaverine, along with two further metabolites. However, tests of the statistical significance of these discriminatory pools were largely limited to a non-parametric univariate test (the Steel–Dwass test, *p* < 0.001, <0.01 and <0.05 for pools (1), (2) and (3) respectively), and it also appears that precautions for FDR corrections were not taken. A similar approach yielded 28, 48 and 27 discriminatory metabolites for breast cancer, pancreatic cancer and PD, respectively (however, the *p* value obtained for such differences was only <0.05). 

Additionally, multiple logistic regression models applied gave a high area under the receiver-operating characteristic curves (AUROCs) for the distinction of each disease category from the healthy control group; these values were 0.865, 0.973 and 0.993 for oral, breast and pancreatic cancers, respectively, along with 0.969 for PDs. 

Qualitative pathway topological analysis (*MetaboAnalyst 5.0,* https://www.metaboanalyst.ca, (accessed on 19 August 2022), University of Alberta, Edmonton, Canada) performed on each of these three pools of biomarkers by the authors of the current study revealed that only the first set tested with 14 named biomolecules and the very highest level of significance (*p* < 0.001) implicated only the branched-chain amino acid (BCAA) catabolism and histidine metabolism pathways as being perturbed in oral cancer patients; however, although the raw *p* values for these pathways were highly significant (4.76 and 8.46 × 10^−3^, respectively), they did not remain so following FDR correction (*p* = 0.13 and 0.18, respectively). 

Interestingly, downregulated concentrations of all three BCAAs, together with that of alanine, have been demonstrated in pancreatic cancer tissue biopsies using solid-state ^1^H MAS NMR analysis [22]. Similarly, low contents of these BCAAs and that of lysine have been found in breast cancer tissue biopsies [19]. These lowered amino acid concentrations may arise from an accelerated level of energy metabolism, or an enhancement of their biosynthetic pathways, along with the mandatory proliferation of cells in cancer tissues. Nevertheless, in Ref. [21], the saliva levels of these amino acids were higher in a series of cancer patient groups than they were in healthy controls. Therefore, there appears to be a major dysregulation in salivary-blood-cancer tissue equilibria and distribution for these amino acids. The authors of the results presented surmised that this observation was ascribable to the heterogeneous systems responsible for the salivary gland transport of amino acids from blood to saliva; for example, differences in the rate of transfer, or the influence of small ions, e.g., Na^+^ and K^+^ [23], the concentrations of which may be modified in view of the passage of water via the paracellular course [24], or channels featured [25]. Nevertheless, salivary gland metabolism may also significantly contribute towards differences observed between the salivary and blood or salivary and cancer tissue biopsy metabolomes. As noted in Ref. [1], such comparisons are markedly complicated by the prominence of a major principal component ascribable to microbial metabolism, in addition to one arising from the host alone. Therefore, as noted above, additional validation of these observations made by seeking further inter-relationships between the metabolic profiles of saliva with those of blood and afflicted tissue samples is required in order to further our understanding of these differences. Of further relevance, in 2007 Yang et al. [26] reported a new approach for comparative metabolome analysis with a view to gaining key information regarding the involvement of metabolite pools and fluxes related to essential metabolic pathways in both model healthy and cancer disease mammary epithelial cell lines. This study involved the tracking of the ^13^C label in ^13^C-labelled glucose using 2D NMR and GC-MS analysis featuring an isotopomer modelling strategy. These researchers found significant differences between the two cell lines which were concordant with previously documented effects, including upregulations in the biosynthesis of FAs. Additional modifications were also observed, and these, according to the authors, for the first time revealed an astounding mileux of ‘global metabolic rewiring’ in the cancer cell line evaluated. 

## 4. Oral Cancers

Oral cancers (OCs) are classified as oral cavity malignant tumours and represent the sixth most common forms of cancers globally, with an incidence of 400,000 new cases per annum, which account for 4% of cancer conditions in men, and 2% of those in women. A series of potentially malignant oral conditions (PMOCs) usually precedes OC onset. These comprise oral submucous fibrosis (OSMF), oral leukoplakia (OLK) and oral lichen planus (OLP); malignant conversion of these PMOCs to OC has incidences ranging from 2 to 30% [27,28]. 

Very recently, Patil and More [29] conducted a systematic review of 10 publications (38 excluded) for the use of salivary metabolomics for diagnosing OC and its preconditions. From this review, metabolic biomarkers found included 1-methylhistidine, 2-oxoarginine, norcocaine nitroxide l-isoleucine and γ-aminobutyryl-lysine l-homocysteate, polyamines (amino acid metabolism); sphinganine-1-phosphate and galactosphingosine (sphingolipid metabolism); 2-phosphoglycerate (carbohydrate metabolism); pseudouridine (nucleotide biosynthesis pathway); 4-nitroquinoline-1-oxide, ubiquinone and reduced glutathione (oxidative stress pathway); estrone-3-glucuronide and estradiol valerate (estrogen metabolism); inositol-1,3,4-triphosphate (electron transport chain); choline, S-adenosylmethionine and methionine (quaternary amine metabolism); BCAAs (TCA cycle, BCAA degradation); urea (urea cycle); and the ketone bodies 3-D-hydroxybutyrate and hydroxy-isovalerate (lipid metabolism).

Although all are ^1^H NMR-detectable, only some of these agents are quantifiable in WMSS specimens using this technique (specifically BCAAs, 2-methylhistidine, specific polyamines, choline, methionine, urea and selected ketone bodies) because of some restrictive sensitivity limits, although this fraction of the above biomarker analytes may indeed be sufficient for diagnostic purposes using metabolomics technologies. The authors of Ref. [29] concluded that the salivary biomarkers found arose from perturbations to pathways involved in the metabolism of amino acids, proteins, carbohydrates and nucleic acids throughout multistage carcinogenesis developments. Indeed, literature data available were found to identify apparently ‘unique’ metabolite signatures characteristic of OC and PMOCs. However, as is nearly always the case, differences observed between the investigative techniques employed in the studies evaluated served to complicate this systematic review, i.e., there were at least some major inconsistencies in the methods employed, and therefore a common metabolic pattern remains unrecognised. 

In 2019, Chen and Yu [30] conducted a summative assessment of the latest progress made with the recognition of disease-specific metabolic patterns observed in saliva, in addition to serum and tumour tissues, in cases or oral cancer. Indeed, they concluded that future studies of these conditions should be focused on the establishment of a regimen for the metabolomics profiling of intracellular metabolites in order to characterise any abnormal patterns of such biomolecules present in tumour cells, and also to explore the potential metabolic effects of administered chemotherapeutic agents thereon. 

An additional investigation [31] explored the capacities of the neural networks, logistic regression, and stochastic gradient descent techniques coupled with ten-fold cross-validation approaches to distinguish between the salivary metabolic profiles of periodontitis and oral cancer patients. Overall, this study featured data mining, metabolic pathway analysis, and the investigation of metabolite–gene interaction networks, and the researchers involved discovered that a deep-learning neural network model linked with the TensorFlow program generated the best results, with an accuracy of nearly 80%. Hence, such methods were valuable for the recognition of biomolecular differences found between oral cancer and periodontitis patients.

One quite unusual study by Supawat et al. [32] was focused on characterisation of the biomolecular profiles of whole unstimulated saliva samples collected from oral cancer and healthy control participants using fluorescence, electronic absorption and ^1^H NMR spectroscopies, and to the best of our knowledge, this is one of the first times that simple spectrophotometric analysis has been utilised in a salivary metabolomics context. The ^1^H NMR aspect of this research demonstrated that salivary trimethylamine N-oxide and glycine concentrations were significantly higher in oral cancer patients than in healthy controls. Moreover, the autofluorescence emission and synchronous absorption spectra of saliva were found to differentiate between oral cancer patients and heathy controls (a total of six fluorophores were detectable in human saliva samples). Indeed, significant differences found between the electronic absorption spectra of saliva samples were found to be concordant in terms of zero-order intensities, and the 1st- and 2nd-derivative spectral profiles acquired. However, Lohavanichbutr et al. [33] found that the salivary concentration of glycine, and also proline, were significantly downregulated in saliva samples collected from OSCC patients.

### 4.1. Oral Squamous Cell Carcinoma

Oral squamous cell carcinoma (OSCC) and oropharyngeal squamous cell carcinoma (OPC) are amongst the most common cancers worldwide and are associated with high mortality and morbidity. This area is indeed a very active area of research and represents 90% of all oral malignant neoplasms. These disorders were investigated by Alves et al. in 2021 [34], and for this purpose, the salivary metabolic profiles of 27 OSCC patients and 41 healthy controls were investigated using a GC-MS technique. Overall, they found 24 metabolites with AUROC values >0.80, and with a threshold limit of 0.90, malate, maltose, protocatechuate, lactose and 2-ketoadipate, along with catechol metabolites, were expressed as significant biomarkers for this condition. From these results, disturbances to the malate–aspartate shuttle, β-alanine metabolism and the Warburg effect pathways were identified. As noted by the authors of this report, additional research investigations featuring larger populations should be conducted in order to verify these results. 

In one further key metabolomics study, aqueous ^1^H NMR analysis at an operating frequency of 800 MHz was employed in conjunction with targeted aqueous LC-MS/MS, and global aqueous and lipidomics platforms using LC-Q-TOF techniques to identify biomolecules with the capacity to discriminate between patients with OCC/OPC and healthy controls, and also to potentially differentiate between OCC patients with and without nodal metastasis [33]. However, these researchers employed ordinary linear regression analysis in order to adjust for demographic variables such as age and race, and experimental batches. This procedure is not to be recommended unless it is clear that there are at least relatively strong linear relationships between such variables, and no evidence for this was presented. Indeed, in many clinical and biological studies, many possible relationships between all variables incorporated into studies may be curvilinear, quadratic or even sigmoidal, rather than linear. However, use of the log_2_-transformation as employed by the authors of this paper may serve to convert some non-linear relationships to linearity. Irrespective of such complications, these researchers found that that both proline and glycine differed ‘significantly’ between the OCC and healthy control groups for discovery and validation datasets (although *p* values were only <0.10 after employing an FDR correction). However, no significant differences in mean salivary levels between these test groups, nor between OCC participants with and without nodal metastasis, were discovered. Nevertheless, glycine, citrulline, proline and ornithine concentrations were found to be related to the early-stage OCC condition, although the authors concluded that further investigations were required to confirm these observations for the development of reliable salivary biomarkers for this disease. 

Very recently, Costa et al. [35] explored means of seeking combinations of biomarkers for the diagnosis of OSCC using a novel data mining approach, and which purported to be one of the very first studies to employ advanced data mining techniques for the diagnosis of this malignant neoplasm. However, for this purpose they used a random forest (RF) classification algorithm system, which is actually well known to many metabolomics researchers, including those focused on multicomponent salivary analyses, e.g., [1]. Moreover, ‘state-of-the-art’ computational intelligence/data mining strategies have already been applied to the diagnosis of human diseases, as reported in [36]. Results acquired from this study revealed that glucuronate, malate and, strangely, butyl alcohol were effective in classifying OSSC disease, with a MV area under the curve (AUROC) parameter of 0.91. The authors concluded that the methodology applied was valuable for the discovery of diagnostic biomarkers for diseases other than OSCC and could therefore provide valuable chemopathological and monitoring information for healthcare professionals. 

In 2016, Mikkonen et al. [37] conducted a systematic review to explore how the capacity of salivary metabolic profiles may furnish researchers with valuable chemopathological data regarding an early diagnostic overview of metabolic dysfunctions linked to either OC or PDs. For this purpose, a MEDLINE search using “salivary metabolomics” as a keyword generated a total of 23 results, of which 7 of these were excluded since they were reviews or published as ‘letters to the Editor’. The remainder served as valuable contributions towards this review. Notwithstanding, although already apparent to many researchers, this study discovered a range of experimental challenges, such as those regarding an insufficient understanding of complex metabolic pathways associated with the differing classes of oral diseases investigated. The authors concluded that the salivary metabolomics approach may serve to yield important information regarding the identification of both local and systemic disorders, the former including oral cancers, and may also facilitate the design and refinement of suitable therapeutic strategies. This review also discussed clinical viewpoints on the future potential of salivary metabolomics.

In a further study, Ishikawa et al. [38] studied the influence of period limits following meal consumption for the collection of saliva samples for the identification of oral cancer with metabolomics approaches. Saliva was collected from oral cancer patients (n = 22) either 12 h. following a dinner meal, and at 1.5 and 3.5 h. subsequent to breakfast, whereas healthy control subjects (n = 44) fasted for >1.5 h. before sample collection. Capillary electrophoresis coupled with a mass spectrometric detection system was employed for the determination of hydrophobic metabolites. Overall, it was found that a total of 51 biomolecules differed significantly between these two classification groups at the 12 h. fasting time-point (*p* < 0.05); however, only 15 and 10 metabolites were significantly different at the 1.5 and 3.5 h. time-points, respectively. As expected, AUROC values for this discrimination were found to be highest at the 12 h. fasting time point. From this work, the authors involved concluded that the 12 h. after dinner fasting time-point was optimal for saliva sample collection, and we fully agree that such lengthier or prolonged fasting/oral abstention periods, and perhaps further controls, are absolutely necessary in order to achieve unbiased evaluations of cancer biomarkers, and hence reliable, interference-free estimates of their salivary concentrations.

**Figure 3:**
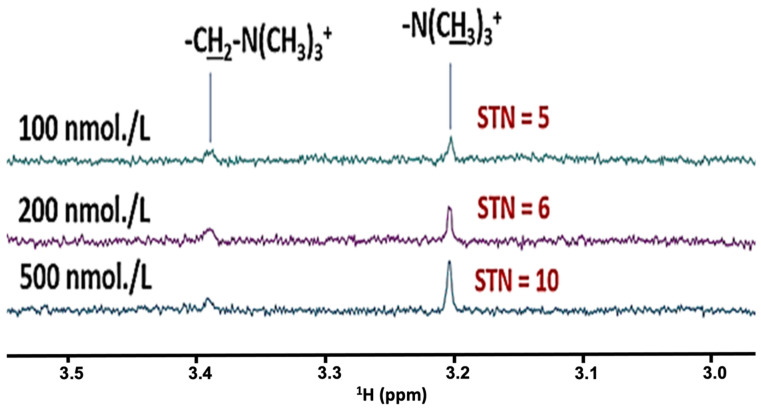
Sensitivity of ^1^H NMR Analysis for Salivary Metabolomics Experiments. Partial (3.06–3.56 ppm regions of) 600 MHz ^1^H NMR spectra acquired on phosphate-buffered (10.00 mmol./L, pH 7.00) aqueous solutions of choline chloride (only 100, 200 and 500 nmol./L). Spectra were acquired on a Jeol JNM-ECZ600R/S1 600 MHz spectrometer operating at a frequency of 600.17 MHz for ^1^H, with a probe operating temperature of 25 °C. Solutions also contained 10% (*v*/*v*) ^2^H_2_O, 330 µmol./L TSP as a chemical shift reference and quantitative internal standard, and 0.03% (*w*/*v*) sodium azide as a microbicide. Spectra were acquired at an operating frequency of 600.17 MHz and 298 K, and with suppression of the very intense H_2_O/HOD resonance (δ = 4.80 ppm) by use of the WASTED-II pulse sequence. Pulsing conditions were: sweep width 11,218 Hz; 16,384 datapoints; acquisition time 1.81 s; relaxation delay 1.00 s; and 1024 transients. STN values for each spectrum were estimated using Jeol Delta-5 software.

These results are fully consistent with our previous investigations [1], which have reported the stringent requirement for the establishment of a minimum abstention period from all possible interfering oral activities before WMS samples are collected, most especially meal consumption. Strikingly, our experiments confirmed that an absolute minimum of 2–4 h. was required to circumvent problems arising from the interference of dietary agents, and this was in marked contrast to recommendations made in other reports that a duration of only 1.0 h. was adequate for this purpose [39]. This is clearly explicable by dietary constituents, e.g., carbohydrates, lipids and organic acids/anions such as citrate, acetate and succinate, etc., clearly persisting in WMSS samples well beyond this 1.0 h. time limit. Additionally, substantial increases in the salivary concentrations of lactate, acetate and other fermentation products were food-induced immediately after eating. Perhaps the application of even more sensitive bioanalytical methods and techniques will establish that this 2–4 h. time restriction should be extended further, for example up to 8–12 h., a duration which our research group now commonly utilises for salivary metabolomics investigations. However, it should be noted that we find that current high-resolution ^1^H NMR spectrometers can extend to the detection of sub-micromolar quantities of salivary biomolecules (Figure 3), and signal-to-noise (STN) ratios of 10 are achievable at analyte concentrations of ≤1 μmol./L for selected analytes, particularly those with proportionately prominent singlet (or doublet) resonances arising from biomolecule -CH_3_ groups, e.g., those of choline, acetone and dimethylsulphone, and a little less so for acetate and creatinine, etc. 

Indeed, Figure 3 shows that for choline’s -N^+^(CH_3_)_3_ head group signal at pH 7.00, a STN ratio of 10 is achievable at a concentration of 500 nmol./L, whereas a value of 5.0 was obtained at a level of only 100 nmol./L; these parameters were determined from spectra acquired with 1024 scans using the WASTED-II pulse sequence, and are very impressive indeed. On consideration of the numbers of protons giving rise to these signals, in principle we may extrapolate and deduce corresponding sensitivity criteria and STN values for three-proton methyl groups which have singlet resonances, e.g., STN ratios of 10 should be achieved in, for example, acetate and pyruvate metabolites, when present at analytical concentrations of only 1.50 µmol./L. So that readers are aware, in our laboratory, we set STN values of 3 and 10 for detection and quantification threshold limits for ^1^H NMR analysis, and therefore a STN value of ≥10 is sufficient for quantification purposes, albeit those ranging from 3–10 are acceptable for signal detection, but not for quantitative NMR analysis. Increases in the sensitivity of this technique are expected with substantial enhancements of the number of scans made within each spectral acquisition, where the STN ratio increases proportionally with the square root of the number of scans made, although one disadvantage of this approach is the quite lengthy acquisitional duration.

Panneeselvam et al. [40] provided a narrative review of the development and implementation of screening tests for the early detection of cancer using approaches which are of minimal invasiveness. This review outlined the development and potential of salivary metabolomics for the discovery and validation of biomarkers for oral cancer diagnosis. In addition to currently available screening technologies in both India and Japan, the prevalence and epidemiologic attributes of and risk factors for oral cancers were considered in detail. Results acquired indicated that the development of biomarkers by itself, per se, is not sufficient for cancer detection and diagnosis. 

Since investigations focused on the applications of metabolomics, NMR-based or otherwise, for the detection of OC using saliva as a biomarker base, it is, of course, necessary to achieve rigorous clinical validation, along with the implementation of standard operating procedures (SOPs) for testing programmes conducted with this biofluid. However, it was suggested that optimal screening programmes should involve a combination of both conventional and newly developed technologies.

Previously, it was demonstrated that an opportunistic screening system for OC was more successful for the diagnosis of precancer and cancer patients than that involving a counter-measure screening protocol [41]. Moreover, a quite recent systematic review performed with accompanying meta-analysis found that the diagnostic accuracies of commonly employed OC screening tests, including standard oral examination, vital rinsing, light-based detection and mouth self-examination, together with remote and biomarker-based screenings, was not reliable for the efficient detection of OC [42]. Furthermore, the design and development of novel technologies for objective evaluations of OC risk is now considered to be critical, most especially because screening conducted by trained dentists and oncology specialists is very costly indeed. To date, a number of machine learning-based data processing and analysis strategies have been established, and these include OC identification, automated disease progress staging, and the application of image processing to distinguish between cancerous and precancerous cells [43,44]. 

## 5. Extra-Oral (Systemic) Cancers

In contrast to oral cancers, breast and pancreatic tumours are physically located remotely from the oral cavity, and therefore one key question is: why exactly should salivary biomolecules serve to indicate their divergent tumour metabolism? To date, evidence is available that salivary biomolecule profiles may indeed provide valuable information on both systemic and localised tumour status or progression, or their responses to chemotherapeutic agents. For example, such methods for indirectly assessing lung and breast cancers have been developed for some time now [21,45,46,47,48,49]. Blood and lymph fluids, as systemic biofluids, serve as avenues which may avoid such tumours, and the salivary gland acts to infiltrate saliva with a blood contaminant. Selected tumour tissue metabolites, e.g., lactate (which arises from tumours exposed to excessive levels of hypoxia), have been found to be upregulated in conditions associated either with or without metastasis occurrence [50,51]. Notably, diminished blood plasma arginine concentrations have also been observed in breast cancer patients. Moreover, abnormal arginine concentrations have also been found in a pooled group of colonic and pancreatic cancer patients, both with and without metastasis [52]. More recently, Hu et al. found an association of plasma arginine concentrations with breast cancer molecular subtypes in women from a North-Eastern Chinese province [53]. 

Furthermore, in OSCC patients without primary tumour metastasis, characteristic cancer-induced modifications to blood serum and salivary mRNA levels have been discovered [54,55], along with alterations to the blood metabolome [56,57]. However, this does not prove that disturbances to the salivary metabolite balance arise from a tumour located remotely. Notably, some previous investigations have revealed upregulations in in blood choline metabolites in a range of cancerous conditions, and in oral cancer patients, elevations in such choline metabolites provided evidence that their passage from blood to saliva through the salivary gland route was low, despite the detection of high blood levels. However, diffusion of choline metabolites from the oral tumour to the salivary gland through an alternative mechanism remains a possibility. Although previous studies have followed metabolomics and data mining protocols to determine whether changes to the salivary metabolome featured cancer-specific characteristics, future investigations should be targeted on comparisons of the complete metabolic profiles of blood plasma/serum and cancer malignancy biopsies with that of saliva in order to recognise any biomolecular associations, including those from cancer-induced metabolic pathway imbalances or malfunctions. 

Notwithstanding, the potential value of salivary biomarkers for the diagnosis and severity monitoring of systemic diseases has been somewhat undermined in view of the lack of physiological and mechanistic reasoning regarding why exactly diseases based at locations remote from the oral cavity could give rise to the development and detection of distinguishing biomarkers in human saliva. In this section, such developments and reasonings are explored further.

### 5.1. Head and Neck Squamous Cell Carcinoma

A recent study [58] explored the ability of salivary NMR analysis for the detection of metabolic modifications putatively arising from the impact of head and neck squamous cell carcinoma (HNSCC). Unstimulated whole-mouth saliva samples collected from HNSCC patients with primary tumours located either in the oral cavity or the larynx, and corresponding healthy controls, donated WMS samples for ^1^H NMR evaluations. Univariate analysis revealed that salivary fucose and propane-1,2-diol were both significantly upregulated in HNSCC patients, whereas the amino acid proline was found to be downregulated. However, it should be noted that propane-1,2-diol is a common exogenous agent present in toothpastes, medications, cosmetics, foods and even cigarette smoke, and therefore an external source of it may be responsible for its detection in WMS and cannot be ruled out. According to the authors, WMS was collected according to a ‘standardised technique’ described in Ref. [59], but no further details were made available on this process, and hence we are unable to deduce whether satisfactory periods of fasting or oral habit abstention were instigated by the researchers involved in this study. MV analysis, however, provided evidence that a composite of four salivary metabolites (fucose, glycine, methanol and proline) was required to achieve a maximal level of distinction between the HNSCC and healthy control cohorts (correct classification rate 92%, sensitivity 87.5% and specificity 93%). Interestingly, fucose has been implicated as a blood serum biomarker for the early detection of various cancers [60,61]. From this work, it was concluded that the human salivary metabolome was significantly responsive to metabolic modifications induced by HNSCC disease. Notwithstanding, exogenous sources of salivary methanol, such as diet and tobacco smoking, remain a complication, and hence further experiments should be conducted to confirm its role as a significant biomarker, along with potentially exogenous sources of propane-1,2-diol. 

More recently, the salivary metabolic profiles of n = 10 head and neck cancer (HNC) patients and 9 primary Sjorgen’s syndrome (pSS) patients, together with 10 healthy control participants were evaluated with a high-performance liquid chromatography-high resolution mass spectrometry (HPLC-HRMS)-based metabolomics technique, as reported by Hynne et al. [62]. From this study, PCA confirmed differential metabolic profiles between these groups, with both HNC and pSS groups showing upregulated ratios of selected pyrimidine nucleotides and nucleosides over those of the corresponding controls; these results suggested that in dry mouth disorders, purinergic signalling may play a key role. Moreover, these researchers also revealed a dysregulation in amino acid metabolism between the groups compared. Indeed, higher salivary concentrations of DL-3-aminoisobutyrate, which is both a terminal purine and BCAA catabolite, were found in both the HNC and pSS groups. Therefore, such metabolic differences found by these researchers should be further explored. 

### 5.2. Lung Cancer

Unfortunately, lung cancer has a high incidence rate. Mutations identified in the EGF receptor (EGFR) represent tumour-specific biomarkers for non-small cell lung carcinoma (NSCLC). One previously noted study performed [63] utilised a novel core technology known as electric field-induced release and measurement, which involves a multiplexible electrochemical sensor, for the detection of EGFR mutations in human saliva, and this approach was shown to be effective, accurate, rapid and cost-effective for the detection of EGFR mutations in this biofluid collected from patients with NSCLC. Additionally, in this study Xiao et al. [63] discovered 16 candidate proteins that had the ability to distinguish lung cancer patients from healthy control participants, and which serve as useful biomarkers for lung cancer with high levels of both specificity and sensitivity. This study revealed that effective proteomic biomarkers can be sought and found in human saliva for the early detection and prognostic screening of lung cancer. In 2012, this group were also successful in establishing a lung cancer-specific transcriptomic biomarker signature in this biofluid [64].

Furthermore, Li et al. [65] performed an analysis of human saliva samples collected from 21 lung cancer patients and 20 healthy controls using surface-enhanced Raman spectroscopy (SERS), and they found that many of the Raman band intensities observed were decreased in the former group. These bands were assignable to proteins and nucleic acids, data which suggested decreases in the salivary concentrations of such agents, although clearly the technique utilised offered only a limited level of molecular selectivity and specificity (although the authors specified that ‘some’ of the bands observed were assigned to certain structural units present in these biomacromolecules). However, PCA and linear discriminant analysis LDA achieved a modicum of success in distinguishing between these two groups, although the accuracy of this application was only 80%.

One additional study [66] focused on distinguishing between the salivary metabolic profiles of patients with lung cancer and those with benign lung lesions (BLLs), and for this purpose 41 and 21 saliva samples, were collected from these groups, respectively, which were analysed using capillary electrophoresis-mass spectrometry (CE-MS). Data were analysed using a multiple logistic regression (MLR) model. These researchers found that a total of ten salivary metabolites substantially differed between these two groups, with tryptophan concentrations being significantly lower in lung cancer patients. However, overall, the AUROC value for this model was only 0.66 (95% CI 0.52–0.81), so it was only barely statistically significant (*p* = 0.036). Further information provided by the researchers involved was that lysine, tyrosine, diethanolamine and cytosine were selected as significant biomarkers when using a back-selection regression option of the MLR analysis; notably, the discriminatory model developed from only these four metabolites yielded an AUROC value of 0.73 (95% CI 0.60–0.86) with a *p* value of 0.003, so this alternative system offered an improved discriminatory potential. In conclusion, the authors suggested that the above four salivary metabolites may find value as potential non-invasive, pivotal biomarkers for discriminating between lung cancer and BLL patients. 

A combination of high-performance anion-exchange chromatography with pulsed-amperometric detection (HPAEC-PAD) was employed by Ragusa et al. [67] to investigate the salivary metabolic profiles of patients with lung and breast cancers (n = 68 patients in total), and how these were differentiated from those of a healthy control group (n = 34). Interestingly, this study involved hydrolysis of the salivary glycoprotein fraction followed by quantification of the free sugars arising therefrom, specifically fucose, galactosamine, galactose, glucosamine and mannose, by an HPLC-anion method featuring pulsed amperometric detection (HPAEC-PAD). The resulting glycosidic profiles were then evaluated and compared using MV and ROC curve analyses. This approach yielded valuable data concerning differential patterns of these sugars between both groups, and which, according to the authors, was sufficient to discriminate between the healthy and cancer-positive groups. 

These observations are potentially of much importance, since glycans are critically involved in signalling, cell–cell adhesion and recognition processes in vivo, and abnormal protein glycosylation patterns have been discovered in a range of pathological mechanisms, including tumour development and progression. Indeed, a number of highly glycosylated proteins such as CA125, CA19–9 and PSA are currently employed as cancer biomarkers in clinical practice. 

### 5.3. Breast Cancer

In 2017, Porto-Mascarenhas et al. [68] performed a systematic review focused on the detection and quantification of salivary biomarkers that may be valuable for the characterisation of breast cancer. Of 567 relevant investigations, only 13 satisfied the inclusion criteria of assessing the diagnostic potential or related distinguishing attributes of salivary biomarkers for this condition. Moreover, such biomarkers were classified in relation to their possible clinical applications. As expected, strategies employing composite biomarkers for this purpose offered a much-improved capacity to diagnose or predict breast cancer rather than single ones. Agents found to be particularly useful as single markers were the amino acids proline, taurine and valine, which were apparently able to assist diagnosis at the early and advanced stages of breast cancer, the latter showing promising diagnostic test accuracy. Interestingly, all these metabolites are readily detectable in the high-field ^1^H NMR profiles of human saliva [1,2,69], and therefore in principle, this technology could easily be employed for breast cancer screening sessions since it simultaneously monitors these biomarkers. However, only a limited number of such investigations reported the essential bioanalytical criteria of sensitivity and specificity, which were both found to markedly fluctuate, specifically from 50–100% and 51–97%, respectively, and therefore further investigations are required for the approval of these amino acids as pre-validated biomarkers. Overall, these researchers concluded that at the time of their report, there was only a restricted level of evidence available to establish the execution of the above salivary amino acids as diagnostic biomarker probes for breast cancer conditions. Of the studies surveyed, only seven investigations explored and reported specificity and sensitivity [70,71,72,73,74,75,76]. Notably, the salivary biomarkers selected were found to detect the later breast cancer phases more reliably than the earlier ones. Hence, the overall conclusions made in Ref. [68] were that there was only a limited amount of evidence available to confirm the potential satisfactory execution of salivary biomarkers as valuable indicators of breast cancer conditions, although this review did offer some new research directives for consideration. 

A detailed meta-analysis targeted on investigating differences between the salivary metabolic profiles of breast cancer patients and healthy controls was conducted by Koopaie et al. [77] in order to evaluate the diagnostic potential of biomarkers identified. Following consideration of a rigorous inclusion and exclusion criteria, and quality thresholds, this study featured 14 publications containing 121 study units, with a grand total of more than 4000 participants in both the breast cancer-positive and healthy control groups. Analysis was performed using specificity and sensitivity, negative and positive likelihood ratios (NLR and PLR, respectively) and diagnostic odds ratio (DOR), along with AUROC and summary ROC plots and assessments. Clinical utility was determined from Fagan’s nomogram. Overall, results obtained in this study were favourable, with significant AUROC values, and post-test Fagan’s nomogram probabilities of 28 and 72% for negative and positive outcomes, respectively. Furthermore, subgroup analysis was conducted to determine the significance and importance of specificity, sensitivity, DOR, PLR and NLR values linked to mean participant ages (< or >52 years old), type of saliva sample (stimulated versus unstimulated) and biomarker class (i.e., metabolomics-, proteomics-, transcriptomics-/proteomics- and biophotonic reagent free-based), along with nations sampled from. In conclusion, saliva was found to contain non-invasive biomarkers which offered much promise for accurately distinguishing breast cancer from healthy control populations.

### 5.4. Pancreatic Cancer

In 2013, Lau et al. [78] explored the hypothesis that pancreatic tumour-derived exosomes are mechanistically associated with the evolution of discriminatory cancer transcriptomic biomarkers present in human saliva; exosomes represent extracellular vesicles produced by all cell types; exosomes are extracellular vesicles produced by all cells, and serve as advocates of both near- and far-distant cellular communications (typically they convey nucleic acids, proteins, lipids and further metabolites). For this purpose, they developed a mouse model of pancreatic cancer that, through the implantation of a mouse pancreatic cancer cell line (Panc02) into the pancreas of the C57BL/6 syngeneic host, generated distinguishing salivary biomarkers. Intriguingly, inhibition of exosome biogenesis gave rise to the removal of such salivary biomarkers. Hence, results acquired provided evidence that tumour-derived exosomes provide an explicable mechanism by which the evolution of salivary biomarkers for pancreatic cancer can be observed, and perhaps also additional distant systemic diseases also of diagnostic importance.

### 5.5. Prostate Cancer

The circulating oncomiRs from body fluids, MiR-141 and miR-21, serve as two tumour biomarkers [79]. Expression of MiR-141 is significantly upregulated in patients with advanced-stage prostate cancer, although miR-21 is overexpressed during early-stage prostate cancer. Hizir et al. [80] have shown that both these biomarkers are indeed expressed in human saliva, and these may be detected using a nano-graphene oxide-based analysis. Therefore, this development offers potential as a minimally-invasive strategy for the early-stage diagnosis of prostate cancer.

### 5.6. Colon Cancer

Intriguingly, a further ^1^H NMR-based investigation [81] showcased a case report focussed on ^1^H NMR-based metabolomics analysis of biomolecules detectable in parotid saliva (PS) samples collected from a single colon cancer patient, both prior and subsequent to chemotherapy treatment for a one-year duration, this involving XELOX: capecitabine plus oxaliplatin. This analysis was supported by concomitant measurements of blood test cancer antigens, along with that for the thyroid peroxidase antibody (TPOAb). This study provided evidence that ^1^H NMR signals for FAs, acetate, citrate, lactate, formate, N-acetylsugars, tyrosine and saccharide species in PS significantly decreased following chemotherapy, whereas blood TPOAb levels significantly increased, and this latter effect mirrored modifications in the 1.0–3.5 ppm ^1^H NMR spectral region. From the changes observed, the researchers involved concluded that these altered metabolic profiles may provide biomarkers for the clinical diagnosis and prognostic monitoring of human colon cancer.

## 6. Oral Mucositis as a Response to Radiation Therapy

Since one of the most commonly observed adverse effects of radiation therapy applied to patients with head and neck cancer (HNC) is oral mucositis (OM), Yatsuoka et al. [82] surmised that an objective assessment of this condition is an urgent requirement for early and timely interventional treatments. For this purpose, these investigators explored the time-course of salivary metabolite profiles in such patients during radiation therapy, and how they may be altered by the severity of OM. A total of n = 9 patients were investigated in this manner. Prior to commencing radiation therapy, OM severity grade (low or high) was differentiated by the salivary amino acids histidine and tyrosine. Additionally, pre-treatment salivary levels of γ-aminobutyrate and 2-aminobutyrate were found to be higher in the high-grade severity OM group. Despite major requirements for validatory studies, this investigation indicated that selected salivary biomolecules were associated with the highest radiotherapy-associated OM grades observed in HNC patients. 

Of especial interest to this area, exposure of healthy or rheumatoid human blood serum to γ-Radiolysis (5.00 kGy) in an atmospheric O_2_ environment was found to generate reproducible increases in the levels of ^1^H NMR-detectable acetate, which were mainly attributable to the sequential hydroxyl radical (^●^OH)-mediated oxidation of lactate to pyruvate, which was followed by the oxidative decarboxylation of pyruvate by radiolytically-generated hydrogen peroxide (H_2_O_2_) and/or further ^●^OH radical [83]. Also detectable were γ-radiolysis-mediated elevations in the serum concentrations of non-biopolymer-bound, low-molecular-mass biomolecules, e.g., citrate and glutamine; this observation may arise from their mobilisation from protein binding sites by the attack of ^●^OH radicals, superoxide anions (O_2_^●−^) and/or H_2_O_2_ at such molecular locations. Moreover, substantial radiolytically mediated elevations in the concentration of serum formate were also observed, and these predominantly arise from the attack of ^●^OH radicals on biofluid carbohydrates, most especially glucose. Hence, in principle, upregulations in the salivary concentrations of products derived from the oxidative activities of radiolytically generated reactive oxygen species (ROS) towards endogenous biomolecules, for example, acetate and formate, may, at least in principle, be observed in samples collected from cancer patients exposed to such radiotherapy treatment in the oral, head and neck body regions. 

## 7. Case Study: An ^1^H NMR Evaluation of Acute-Phase Glycoproteins in WMSS Samples and Their Possible Applications as Biomarkers for Cancers and Inflammatory Disorders: Potential Interferences from ^13^C Satellites, Low-Molecular-Mass Biomolecules and Salivary Hyaluronate 

In blood plasma or serum, N-acetylated glycoproteins, which are part of the large group of acute-phase proteins (APPs), are directly associated with and characteristic of inflammatory conditions such as inflammatory joint and bowel diseases, amongst many other disorders [1]. Importantly, abnormal glycosylation patterns of proteins represent a hallmark of tumourigenesis, and therefore they have the ability to offer valuable biomarker information for the identification and diagnosis of cancerous conditions. Indeed, aberrant protein glycosylation has been linked to disease progression in breast [84], prostate [85], ovarian [86], lung [87] and hepatocellular carcinomas (HCC) [88]. Moreover, particularly notable is the knowledge that human saliva has been postulated to serve as a valuable source of these species in view of the known high proportion of glycosylated proteins in this biofluid [89,90], which also represent quite a high fraction of those within the salivary proteome. Indeed, in the study reported in Ref. [90], a grand total of 156 N-glycosylated peptides, characteristic of 77 distinctive N-glycoproteins, were detected in all salivary fluids examined, i.e., WMS, and parotid, submandibular and sublingual fluids, with 62 being found in WMS. A quite high proportion of these N-acetylated glycoproteins (40%) were expounded as extracellular protein species. Since alterations in the glycation patterns of these proteins and their concentrations have the capacity to exert significant cellular modifications, both integral and functional, in a range of diseases, research investigations in this field are markedly expanding. Hence, biofluid APPs offer much potential as diagnostic biomarkers, e.g., [91] and [92], but to date their detection and quantitative monitoring in human saliva remains somewhat limited. 

Indeed, this research area is not without its complications. As noted for the glycoproteome analysis of human plasma or serum, such investigations tend to be more intricate than proteome analysis, since glycan oligosaccharides and polysaccharides are constituted from a wide range of both linear- and branched-chain sugar residues, a phenomenon which complicates the molecular structures of such glycoproteins [92]. These carbohydrate side chains are covalently bonded to APP asparagine residues through primary chain N-acetylglucosamine residues. Hence, glycoprotein analysis presents a major challenge, even with the availability of ‘state-of-the-art’ bioanalytical facilities. However, in view of its ready application to the elucidation of problems concerning the solution status, structures, molecular mobilities and dynamics of macromolecules in aqueous solution, in addition to low-molecular-mass metabolites, high-resolution ^1^H NMR analysis serves as a potential means of overcoming these hurdles.

**Figure 4:**
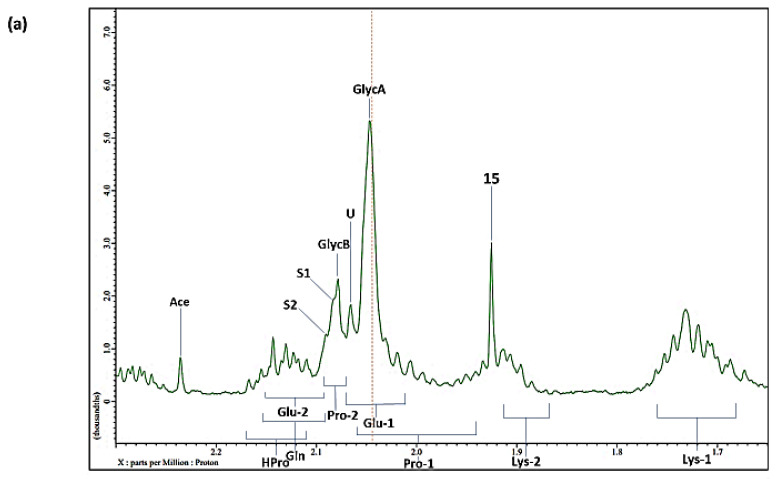

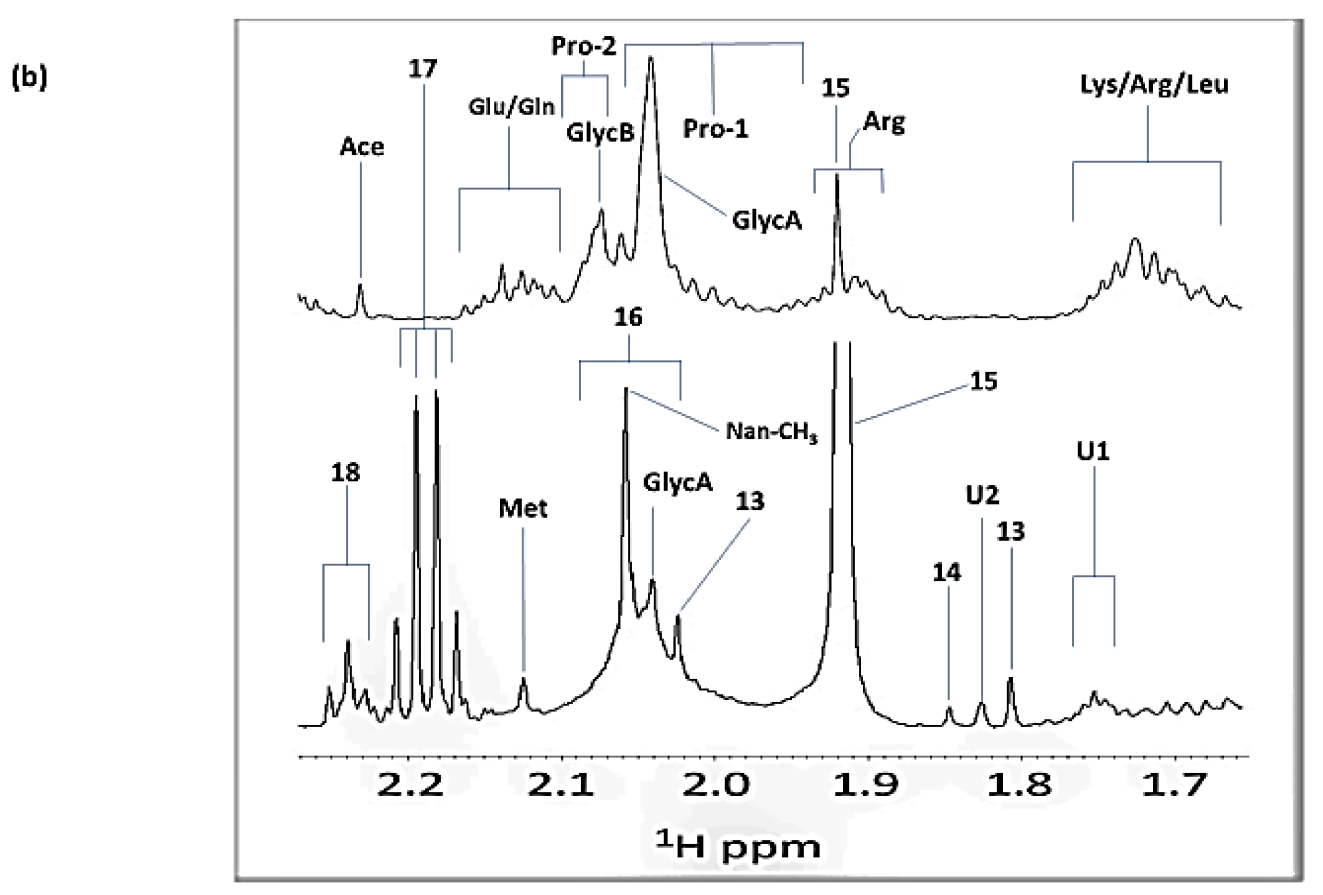
^1^H NMR Analysis of APP Side-Chain N-Acetylsugar Residues Present in Human Saliva and Blood Plasma. (**a**) Expanded 1.65–2.30 ppm region of the ^1^H NMR WASTED-II spectral profile of healthy human blood plasma, showing potential interferences with the quantification of macromolecular GlycA and GlycB species arising from the free amino acids proline and glutamate, and low-molecular-mass N-acetylsugar and possible N-acetylamino acid metabolites. The red vertical line indicates the chemical shift value of free N-acetylglucosamine at δ = 2.044 ppm, which is very similar to that of the much broader GlycA signal. (**b**) Expanded 1.65–2.27 ppm regions of the ^1^H NMR WASTED-II spectra of human blood plasma (top) and WMSS (bottom) samples collected from healthy human participants to allow comparative evaluations of their acetamido-CH_3_ resonances, which arise from both low- and high-molecular-mass biomolecules in these biofluids. Typical spectra are shown. Spectra were recorded on a Jeol JNM-ECZ600R/S1 600 MHz spectrometer operating at frequency of 600.17 MHz for ^1^H, at a probe operating temperature of 25 °C. Assignment abbreviations: As Table 1, with Lys-1 and -2, lysine-γ- and β-CH_2_ groups, respectively; Pro-1 and -2, proline-γ- and β-CH_2_ groups, respectively; Glu-1 and -2, glutamate-β-CH_2_ resonances; Hpro, hydroxyproline-β-CH_2_; Gln, glutamine-β-CH_2_; Met, methionine-S-CH_3_; Ace, acetone-CH_3_; Nan-CH_3_, N-acetylneuraminate-CH_3_; S, S1, U and U1, unidentified resonances, those in the plasma profile shown in (**a**) may being ascribable to further ^1^H NMR-distinguishable -NHCOCH_3_ functions, with S1 possibly representing an N-acetylneuraminate signal in a molecular environment differing from that/those of GlycB; Arg, arginine-β-CH_2_; Leu, leucine-β-CH_2_ and γ-CH resonances.

Although fraught with at least some superimposition problems with other resonances, albeit less-visible sharper ones (Figure 4a), the generalised pattern of acetamido-CH_3_ function glycoprotein resonances in human plasma or serum largely, but not exclusively, comprises two major broad signals, the first a composite of those of *N*-acetylglucosamine and *N*-acetylgalactosamine (now commonly known as GlycA) [93,94], centred at δ = 2.04 ppm, whilst the second, which is located at δ = 2.08 ppm, and known as GlycB, represents terminal *N*-acetylneuraminate residues only [95]. However, further useful functional information on these detectable acute-phase reactants is provided by the ratios H/W GlycA and H/W GlycB, where H depicts signal height, which is related to analyte concentration, and W signal width, which reflects the flexibilities, aggregation status and molecular mobilities of the biomacromolecules giving rise to these distinctive resonances. Notably, both higher and less broad resonances have been associated with the pathologies of a number of inflammatory conditions [95]. 

Overall, the GlycA signal defined for human plasma or serum reflects the integrated levels of the glycosylated forms of circulating acute-phase reactants, largely α1-acid glycoprotein (α1-AG), haptoglobin, α1-antitrypsin, α1-antichymotrypsin and transferrin [95], but predominantly α1-AG. These, in turn, are mediated by multiple cytokine secretions from activated neutrophils [90,91]. With the exception of transferrin, the circulating concentrations of the proteins that constitute the GlycA signal increase during the acute-phase response [96].

Fuertes-Martín et al.’s recently-conducted systematic review [94] concluded that the GlycA acetamido-CH_3_ group resonance(s) acts as a reliable marker of systemic inflammation. Indeed, results acquired have indicated that this GlycA marker captures systemic inflammation more effectively than the more classical and widely employed C-reactive protein (CRP) marker. The authors concluded that GlycA potentially served as a key marker for many human diseases, e.g., cardiovascular and metabolic disorders, and cancer, along with a range of chronic inflammatory diseases. In fact, an upregulated level of the GlcNAc-branching status of *N*-glycans has been found in tumour and cancer patients [94]. 

Interestingly, in 2017 Kianoush et al. [97] explored differences between ^1^H NMR-detectable blood serum GlycA concentrations in former and current tobacco smoking participant groups, along with a never-smoking control cohort, as part of the very extensive Multi-Ethnic Study of Atherosclerosis (MESA), and the Brazilian Longitudinal Study of Adult Health (ELSA-Brasil). The relationship of levels of this NMR-unique biomarker to those of high-sensitivity C-reactive protein were also evaluated. These researchers found that mean serum GlycA levels for current and former smokers (414 and 393 µmol./L) were significantly higher than that for never smokers (391 µmol./L respectively) when the adjusted mean values were tested in an MV analysis model. However, in a univariate context, it should be noted that these differences were actually only 5.9 and 0.3% higher than the never smoker control value, and conceivably these increases observed may indeed be within the remits of experimental error, or more specifically ‘within- and/or between-assay’ analytical reproducibilities, most especially the latter one. Significant dependencies of all forms of smoking behaviour on logarithmically-transformed GlycA and high-sensitivity C-reactive protein levels were also found. 

Figure 4b shows the expanded 1.65–2.27 ppm regions of the 600 MHz WASTED-II pulse-sequence ^1^H NMR profiles of typical healthy WMSS and blood plasma samples to enable readers to make comparative evaluations between resonance patterns therein. However, for human WMSS samples, these broader APP resonances are less well defined than in blood plasma/serum, and it appears that only the GlycA signal centred at δ = 2.04 ppm is visible in the single-pulse spectra acquired (Figure 1). At high operating frequencies, the ^1^H NMR profiles of human salivary supernatants always contain a series of sharper acetamido function (-NHCOCH_3_) resonances within the 1.90–2.10 ppm spectral region, and these typically overlay much broader resonances arising from this functional group in N-acetylsugars located within the molecularly-mobile carbohydrate side chains of APPs. Indeed, these broader resonances remain clearly visible in spectra acquired using spin-echo Carr-Purcell-Meiboom-Gill (CPMG) or the WASTED-II [98] pulse sequence applied here to the ^1^H NMR analysis of human saliva in aqueous media (Figure 1), since these are much more molecularly mobile than their corresponding native apoprotein moieties. However, our factor analysis conducted in Ref. [1] indicates that these glycoprotein species, along with ‘free’ low-molecular-mass N-acetylsugar species, which are also ^1^H NMR-detectable in this spectral range, arise from the host and not salivary microbiome sources in this biofluid. Moreover, the investigations performed by Gardner et al. [99] largely confirm this, since the ^1^H NMR spectral profiles of matched parotid saliva also contain these acetamido function signals (both broad and sharper). Indeed, digital subtraction of the spectra of matched parotid saliva specimens from those of corresponding WMSS samples [99] was found to give rise to the complete removal of these resonances from the spectral profiles. The glycosylation process critically features protein folding and stabilisation, which gives rise to changes in cellular adhesion, antigen recognition, and cell signalling phenomena. Therefore, these resonances may reflect recognised inflammatory states, since their heights, linewidths and overall intensities provide valuable information on not only biofluid levels, but also structural diversifications of their acute phase reactant identities. Of the three (or more) sharper resonances detected in WMSS spectra acquired, that located at δ = 2.028 ppm is clearly assignable to the ^13^C satellite resonance of acetate’s -CH_3_ group, the intensity of which is 0.54% of that of the major, spectrally dominating main ^1^H singlet signal of this metabolite located at δ = 1.92 ppm; the corresponding higher field ^13^C satellite at δ = 1.80 ppm is also clearly discernible in all single-pulse *noesy-presat* spectra acquired (Figure 1). Fortunately, application of the WET pulse sequence effectively extinguishes ^13^C satellites in ^1^H spectra [100], and, as expected, these were eliminated from the spectral profiles when this sequence was applied (Figure 1c,d). 

Of particular interest, mean acetate concentrations in WMSS samples collected from a series of human participants vary markedly, and range from 31 to as much as >300 mmol./L [2], with an overall average value of 108.2 mmol./L. Therefore, the mean intensity of each of its ^13^C satellites, which is 0.54%, would be equivalent to a singlet ‘resonance’ of a -CH_3_ group-containing molecule of salivary concentration 0.58 mmol./L, which is certainly more than high enough to interfere with any determinations of GlycA made. Moreover, if the salivary acetate concentration was only 10.0 mmol./L, this satellite signal would contribute an equivalent level of 54 μmol./L to the ‘apparent’ GlycA signal, again a significantly interfering quantity. 

From Figure 4, it can be observed that resonances arising from the low-molecular-mass forms of glutamate and proline may serve to superimpose on the GlycA resonance of blood plasma, and hence perhaps significantly interfere with its ^1^H NMR determination, and also its width at half-height and height parameters employed for indicating its molecular parameters in the plasma solution state. Nevertheless, proline, and to a lesser extent, glutamine signals, may significantly impact on concentration and molecular mobilisation measurements made from the GlycB resonance intensities and linewidths, respectively. Likewise, these resonances may also interfere with the identification and/or determinations of GlycA, and any GlycB species, which are ^1^H NMR-detectable in the WMSS spectra acquired. 

**Figure 5:**
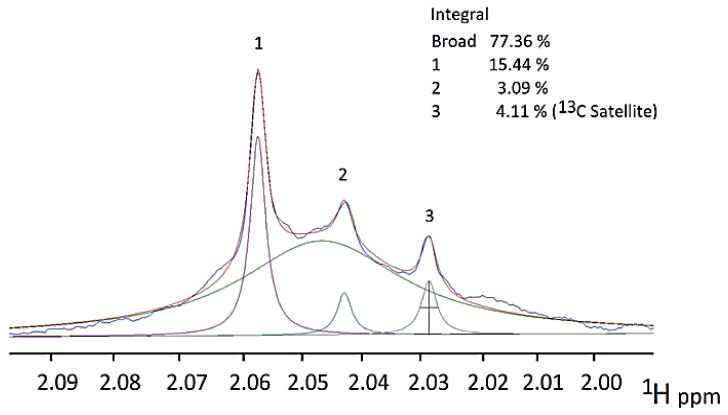
Deconvolution of the ^1^H NMR Resonances of Salivary APP Side-Chain N-Acetylsugar Residues. Deconvolution line-fit of the signals present within the 1.99–2.10 ppm acetamido-CH_3_ group region of the 600 MHz ^1^H NMR profile of a typical human WMSS sample, which was achieved using Jeol Delta-5 software modelling. This deconvolution approach yielded a single major broad resonance (δ = 2.046 ppm) presumably ascribable the GlycA signal detectable in human blood plasma, and three sharper ones located at 2.059, 2.042 and 2.028 ppm (labelled 1, 2 and 3, respectively), which are assignable to free N-acetylneuraminate- and N-acetylglucosamine-NHCOCH_3_ group resonances, and the dominant acetate-CH_3_ group’s lower field ^13^C satellite (confirmed through the acquisition of ^13^C decoupling WET spectra (Figure 1c,d), respectively. The WMSS spectrum was acquired with a Jeol JNM-ECZ600R/S1 600 MHz spectrometer operating at frequency of 600.17 MHz for ^1^H, and at a probe operating temperature of 25 °C, using the *noesy-presat* pulse sequence, although only eight scans were required for this.

Figure 5 shows a software-based spectral deconvolution of the δ = 1.90–2.10 ppm region of the *nosey-presat* ^1^H NMR spectrum of a typical human WMSS sample, which clearly reveals four overlapping signals therein, the most predominant being a broad one (Δ*v_1/2_* = 15 Hz) comprising 77.4% of the entire resonance magnitude in this spectral region. The chemical shift value of this signal, along with its width at half-height and height parameters, are very similar to those of the GlycA resonance commonly found in human plasma (Figure 4a,b). The singlet signals at δ = 2.042 and 2.059 ppm certainly appear to be ascribable to the ‘free’ N-acetylsugars N-acetylglucosamine and N-acetylneuraminate, respectively. Resonances presumably attributable to free N-acetylneuraminate and free N-acetylglucosamine, and acetate’s ^13^C satellite, accounted for 15.44, 3.09 and 4.11% of the intensities, respectively, of all detectable signals within this region. Also notable is the possible superimposition of unsaturated FA (triacylglycerol or otherwise) allylic-CH_2_-CH=CH- signal(s) at the higher-field region (*ca.* δ = 2.02 ppm), but this interference was found to be very small when compared with that observed in human blood plasma [93]. Intriguingly, only eight scans were required to acquire these data at an operating frequency of 600 MHz. 

Table 2 shows results from our own literature review survey of all known ^1^H NMR resonances, including those of potentially interfering endogenous metabolites, appearing within the APP regions of spectra acquired on healthy human blood plasma and WMSS samples. Also provided are estimates of their concentrations in each biofluid from Ref. [101]. These results demonstrate that in addition to the broad unsaturated FA allylic-CH_2_-CH=CH- resonance, which is known to be at least partially superimposed with the GlycA signal in blood plasma [93,94,95], and hence complicates its measurement without the application of acceptable deconvolution techniques, there are also a series of further interferants naturally available in both biofluids. These interferants include the amino acids glutamate and proline. Interestingly, salivary levels of ‘free’ N-acetylneuraminate are quite substantially higher in human saliva than they are in blood plasma, and this may reflect its more ready or rapid release and mobilisation from APP molecularly mobile carbohydrate side chains in the oral environment. 

Since currently there are no available scientific literature data on the salivary concentrations of ‘free’ N-acetylneuraminate and N-acetylglucosamine, deconvolution of their acetamido-NH-CO-CH_3_ signal patterns in WMSS sample spectra, as demonstrated here, may indeed provide a valuable means for determining their concentrations in this biofluid medium. 

Predominately, the ^1^H NMR resonances of the glycoprotein carbohydrate side chain sugar ring protons are unfortunately not readily discernible in blood plasma spectral profiles in view of a substantial level of superimposition with the more intense signals within the 3.00–4.00 ppm regions, notably those arising from relatively high levels of glucose in this biofluid [102]. However, although there remain many metabolite signals in this region of the ^1^H NMR profiles of WMSS samples, resonance ‘crowding’ therein is somewhat less marked than it is in that of plasma. Indeed, one further, albeit tentative, assignment made in the ^1^H NMR salivary profile shown in Figure 1 is that for the -C4H proton of free N-acetylneuraminate (*m*, δ = 4.02 ppm). This corresponds to the more intense sharp singlet resonance assignable to the -NHCOCH_3_ group of its ‘free’ form located at δ = 2.06 ppm. 

However, in addition to mucin-glycoproteins, this acetamido function spectral range of WMSSs may also contain some ^1^H NMR contributions from hyaluronate, a high-molecular-mass glycosaminoglycan with linear repeating glucuronate/N-acetylglucosamine disaccharide units (it comprises between 200–10,000 of these units, and normal tissue molecular masses of it may exceed 10^6^) [103]. These contributions may be ascribable to hyaluronate itself (broad resonance(s)), and/or low-molecular-mass oligosaccharide/saccharide species arising from its depolymerisation by bacterial hyaluronidase [2], or alternatively, through the actions of pathologically mediated ROS such as the aggressively reactive hydroxyl radical (^●^OH) [83,104]. Notwithstanding, mean salivary hyaluronate concentrations in unstimulated whole human saliva (459 ng/mL) are actually very low. Further studies have found that it is present in unstimulated human saliva within the range 148–1270 ng per milligram of protein in unstimulated whole saliva [105,106]. This marked variation appears to be attributable to a wide range of factors, including diet, oral hygiene and anatomy, health and disease status, genetics, plus further explanations.

From the relative molecular masses of glucuronate and N-acetylglucosamine, if we assume that 53% of this level represents the latter sugar, then its salivary concentration would be only ca. 1.0 μmol./L, and therefore this glycosaminoglycan as a whole certainly would not be expected to contribute towards the broader APP signals observed in this biofluid. However, higher, more significant concentrations of N-acetylglucosamine itself may arise from its accumulation through continued depolymerisation or fragmentation processes. 

**Figure 6:**
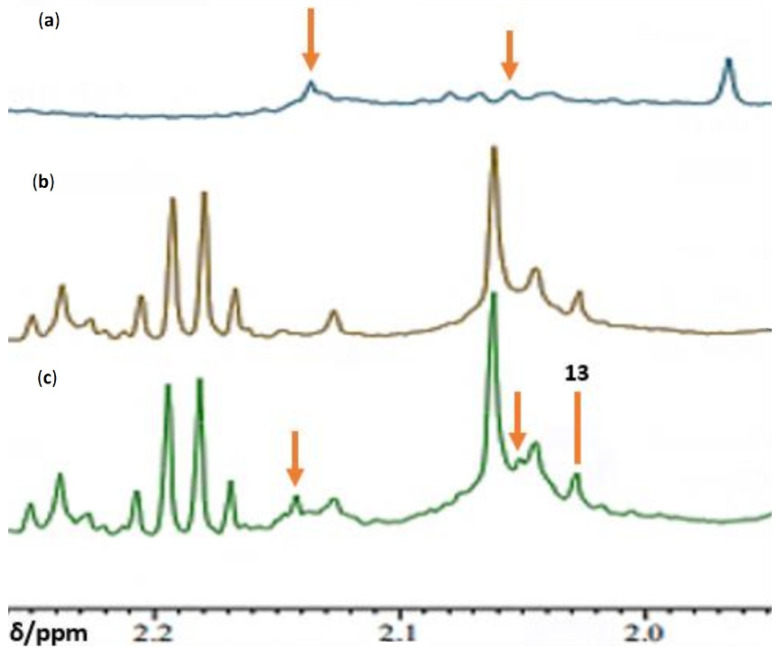
^1^H NMR Evaluation of the Treatment of WMSS Samples with the Enzyme Hyaluronidase. Partial (1.50–2.26 ppm regions of) 600 MHz WASTED-II ^1^H NMR spectra of (**a**) an aqueous solution containing a commercially available hyaluronidase enzyme preparation (4167 units/mL in aqueous solution containing 8.33% (*v*/*v*) ^2^2H_2_O); (**b**) a typical untreated WMSS specimen; (**c**), as (b), but treated with 4167 units/mL of the hyaluronidase preparation (both control and hyaluronidase-treated saliva samples also contained 8.33% (*v*/*v*) ^2^2H_2_O). Both untreated and treated saliva samples were equilibrated for a duration of 8.00 h. at pH 6.50 and 25 °C prior to ^1^H NMR analysis. Assignment abbreviations: as Table 1. The arrows in (**c**) indicate resonances arising from ^1^H NMR-active contaminants introduced from the added enzyme preparation. Spectra were recorded on a Jeol JNM-ECZ600R/S1 600 MHz spectrometer operating at frequency of 600.17 MHz for ^1^H (probe operating temperature 25 °C).

A further experiment conducted by our group involved the treatment of human WMSS samples with the enzyme hyaluronidase in order to determine if any of the broader 1.96–2.10 ppm range signals (and consequently, perhaps one or more of the sharper saccharide sugar/fragment resonances) arose from hyaluronate. As expected, performance of this reaction on authentic hyaluronate samples in aqueous solution at pH 6.50 generated a very intense sharp acetamido-CH_3_ group signal ascribable to free N-acetylglucosamine (δ = 2.044 ppm, data not shown); this sharp resonance has also been shown to arise from the fragmentation of authentic hyaluronate, or in hyaluronate-containing inflammatory human synovial fluid, by radiolytically generated ^●^OH radical [104]. However, equilibration of human WMSS samples with this enzyme under the same experimental conditions failed to liberate any low-molecular-mass N-acetylsugar species, and no sharp resonances within the above spectral range were generated from this process (Figure 6). Therefore, in addition to the above concentration considerations, we conclude that the broader N-acetylsugar-NHCOCH_3_ function signals detectable in WMSSs did not appear to encompass any of those arising from hyaluronate. 

Further valuable reports available in the fields of APPs, their NMR-mobile carbohydrate side chains, and oligosaccharide and/or monomeric sugar residues derived therefrom, include (1) the distinction of glycoprotein ^1^H NMR resonance patterns in rat blood plasma from those of humans, specifically the detection of both O-acetyl- and N-acetylsugar chain residues in rat plasma APP carbohydrates, but only the latter sugars in those of human plasma, a paper which clearly established the species-dependence of the molecular nature of APP sugar chain residues [107]; and (2) the use of high-resolution ^1^H NMR analysis to identify and monitor urinary N-acetylated metabolites, comprising amino acid, mono- and oligosaccharide species, in order to provide valuable biomarker evidence for the monitoring of patients with a variety of inborn errors of metabolism, including Niemann-Pick type C1 and Canavan’s diseases [108]. 

## 8. Clinical Implications of the ^1^H NMR-Based Metabolomics Analysis of Human Saliva for Cancer Detection and Monitoring

Potentially valuable diagnostic and prognostic disease monitoring criteria arising from these investigations may provide opportunities for the development of novel biosensor devices operational in a multianalyte format which will achieve the simultaneous determination of, for example, up to five of the most diagnostically significant biomarkers selected by NMR-based metabolomics platforms. This may indeed yield an invaluable method for the MV evaluation of oral or systemic cancers using saliva as a test matrix at patient points-of-contact (health clinics and medical practitioner practices, etc.). Indeed, such a hand-held biochip reaction platform could realistically incorporate combinations of appropriate immobilised enzymes and reagents required for this multi-analytical process, which jointly serve as detector systems for the quantification of each individual biomarker. Data acquired therefrom can be easily exported to a PC with appropriate chemometrics software for this purpose, if required. Such readily portable hand-held devices will serve as a major diagnostic and perhaps status monitoring benefit for clinicians and supporting healthcare staff. 

Similarly, novel NMR-linked metabolomics-network enrichment ratio/topological pathway analysis has the potential to provide a wealth of information regarding (1) the major roles of featured genes/proteins in oral and/or systemic cancers and their drug-targeting; (2) cellular processes that are likely to be influenced by the inhibition or activation of a target protein, and which of these are therapeutically significant or alternatively give rise to deleterious health effects (specifically the nature of upstream activators or downstream targets for key proteins identified); (3) the ability of multi-bioanalyte ^1^H NMR datasets to inform us of modifications to cellular functions and pathways, together with potential intervention sites for drug actions; (4) the nature and potential activities of key metabolites and pathways involved in human cancer conditions; and (5) the identification and validation of identified biomarkers, which may also be employed for the assessment of drug actions and efficacies. Such clinically important information is clearly of much economic importance to health authorities (and further researchers) in view of the selection and longevity of possible treatments and/or treatment regimens for these diseases. However, as suggested in Ref. [40], the optimisation of drug screening experiments should ideally involve a comparative evaluation of both currently-available and newly-developed NMR-based metabolomics technologies, since this report indicated that the availability of metabolomics data alone may be insufficient for reliable diagnostic purposes.

It is also anticipated that the above research protocol will readily promote the passage of NMR analysis, both high-resolution and conceivably benchtop spectrometers, into the realm of routine chemical pathology investigations for patients afflicted with cancer conditions.

## 9. Routes to Therapeutic Options and Drug Discovery

### 9.1. Recognition of Drug Targets 

In addition to their abilities to successfully evaluate the significance and impact of alterations in metabolite concentration variables engendered by the development, progress and perhaps severity of human cancer conditions (at an extensive multidimensional level), MV metabolomics analysis protocols also offer the ability to reliably build networks for the seeking and identification of suitable drug targets, and also for the detection and critical analysis of drug action pathways [109]. Indeed, composites of such metabolomics and network enrichment analysis strategies allow the confirmation of potential target protein(s)/enzyme(s), a process enabling researchers to design, develop, characterise and apply (primarily in cell culture and animal model system experiments) novel therapeutic agents or measures which have the ability to exert powerful inhibitory (or other therapeutically relevant) activities towards them. The augmentation of predictive models of therapeutic efficacies for drugs with yet unknown or only speculative mechanisms of action can also be facilitated by this strategy. Once established, drug target validation involving determinations of their potentials as critical destination sites for drug action may then be applied. Follow-up validation studies then often involve the performance of additional screening processes to assess and further explore any drug ‘hits’ to the target(s) identified. Moreover, the detection of metabolic modifications arising from progressing disease activities may serve to identify biomolecular perturbations that are pivotal to the pathogenic cascades of cancers, or other diseases, related or otherwise. If targeted in this set-up, particular drug destination sites that are predicted to offer maximal clinical benefits may be identified. 

### 9.2. Drug Discovery Programmes

Results arising from such studies can, at least in principle, also serve as a critical aspect of future drug discovery programmes for human diseases. Furthermore, the successful identification of such drug targets serves to inform researchers on the mechanism(s) of action of newly developed prospective drugs, and also furnish them with a fully comprehensive understanding of their pharmacological role(s). Typically, the ^1^H NMR-based metabolomics techniques outlined in the current study, along with the involvement of further biochemical and bioanalytical methods, where required, may also have the capacity to reconstruct regulatory networks, signalling cascades and/or metabolic pathways as features of future drug development or treatment regimens for selected human diseases, and potentially also for related or associated conditions. 

Although to date proteomic network analyses have emerged as a key tool for probing the nature, specific structural properties and physiological activities of potential drugs, which also act to improve our understanding of the implications of drug-target interactions at the precise molecular level [110], there is also much evidence available to suggest that ^1^H NMR-linked metabolomics analysis strategies also provide much potential concerning the delivery of valuable drug discovery information, together with that regarding mechanisms for their therapeutic actions [111]. Network enrichment ratio and topological pathway analyses are focused on the analysis of groups of metabolites which are involved in or are related to specific metabolic pathways, and such approaches serve to determine the probabilities of selected biomarkers arising from one or more of these sources, and hence their likely involvement in any disease-associated dysregulated pathways, either as a single metabolite disturbance, or more usually as groups of them. Likewise, the physiological location (cellular and sub-cellular, organs, tissues, etc.) and suggested disease manifestations of such imbalanced pathways may also be considered. The molecular nature, and cellular locations of protein targets for potential drugs, serves as a critical primary knowledge requirement regarding drug design and drug actions [112], and network enrichment analysis is valuable for seeking and confirming these. Software employed for such purposes is based on high-quality KEGG and metabolite set enrichment analysis (MSEA) metabolic pathways as the backend knowledge base. 

Recent developments in this area have provided a high level of guidance regarding the identification of novel drug targets via either hypothesis-driven research, or expansive screening protocols. In conjunction with bioinformatics and systems biology approaches, genome sequencing and molecular pathology efforts are continuously refining our level of understanding of exactly how disease-affected cells are ‘programmed’, and hence how exactly they may serve as targets for single drug agents, or synchronously at two or more target locations via drug combination strategies [113,114,115].

Of much relevance to the current study, a large number of major advancements in the molecularly-targeted drug discovery research area have featured those involving small molecule anti-cancer drugs. Indeed, such developments have indeed given rise to an increasing number of successful treatments that have substantially impacted upon the lives of many cancer patients. Notably, the therapeutic administration of anti-oestrogens and anti-androgens to treat hormone-driven breast and prostate cancers is now well established. Additionally, the highly effective, curative therapeutic activity of all-*trans* retinoic acid for treatment of a high proportion of patients with acute promyelocytic leukaemia who harbour translocations in the retinoic acid receptor (RAR) α gene have, to date, implemented validity of the perception of clinically-targeting pathogenic driver abnormalities with small molecule therapeutic agents [116]. Moreover, the Abelson tyrosine kinase (ABL) inhibitor imatinib is a ground-breaking drug that has most drastically validated the notion of designing low-molecular-mass molecules as therapies available to treat pre-specified patient populations, i.e., chronic myeloid leukaemia, in which the malignancy is driven by the BCR-ABL translocation, and for which improvements in survival rates have been very impressive indeed [117,118].

These resounding successes have been followed by the employment of further low-molecular-mass drug molecules targeted at the suppression of critical cancer targets. These include the epidermal growth factor receptor (EGFR) kinase inhibitors gefitinib and erlotinib, which strikingly inhibit this receptor in non-small cell lung cancer (NSCLC) patients; the EGFR/ERBB2 inhibitor lapatinib for ERBB2-positive breast cancer; and the vascular epidermal growth factor receptor (VEGFR) kinase inhibitor sorafenib for the treatment of renal cancer [119].

Such examples provide a high level of evidence for the successes achieved with the ‘targeting’ of metabolic profile and pathway imbalances which have roles as pathogenic ‘drivers’ in human diseases. Of major interest to the current study, the ‘oncogene addiction’ process (also known as the ‘Achilles heel of cancer’) also readily provides an appropriate rationale and justification for the use of molecular-targeted therapies for the treatment of pre-selected cancers [120,121].

### 9.3. Potential Facilitation of Decisions to Be Made on Drug Treatments in Oncology (Untargeted or Targeted) with Available Metabolomics Datasets 

Hence, in principle, these approaches may play major roles in the design and development of therapeutic agents for cancer treatment, either for local oral or distant systemic cancers. Currently, chemotherapeutic agents most commonly utilised for oral cavity or oropharyngeal cancers are cisplatin, carboplatin, 5-fluorouracil, paclitaxel, docetaxel and hydroxyurea. However, all of these agents exhibit some quite serious adverse side effects, especially when two or more of these agents are used in combination. 

Nevertheless, in the context of clinical epidemiology (Section 1.3), ideally all such chemotherapeutic agents should firstly be evaluated in terms of their interventional actions, and therefore NMR-linked metabolomics analysis may serve to offer some clinical benefits here, although any prior information on whether or not the mechanisms of action of these drugs are known, and/or whether any known or potential drug targets for them have already been elucidated and explored (for example, primarily DNA for cisplatin and carboplatin, but also RNA as a secondary target, along with a range of specific proteins [122]), would facilitate decisions on drug selection and dose. Such considerations may also involve a detailed critical analysis of the scientific and clinical literature (including systematic reviews, MV meta-analyses conducted, etc.), so that the most efficient therapeutic options may be evaluated. ‘State-of-the-art’ metabolomics technologies, including biomarker recognition, may further facilitate these decisions through their abilities to seek and inform on the therapeutic actions of these agents in relevant cell culture experiments, or alternatively biofluids and/or tissues collected in animal model system investigations, and ultimately those from cancer patients themselves before and after receiving these treatments. Such studies should involve a full scrutiny of the actions of chemotherapeutic agents to clinically intercept pathological mechanisms of the diseases explored. However, the identification of drug risk (side effect) factors, and also any prognostic and protective criteria, and drug resistance issues, should also be critically considered.

Therefore, in principle, ^1^H NMR-linked metabolomics analysis of human saliva samples, particularly WMSSs, can also serve to provide much valuable information regarding biomarker identification and validation, the pinpointing of dysregulated metabolic pathways and the recognition of potentially useful drug-targeting sites on malfunctioning proteins/enzymes. Nevertheless, it is of much importance to note that unless the cancer class is oral in locality, or it has the ability to indirectly but significantly influence the salivary microbiome or metabolome from a remote source, then such proposed investigations may be severely limited for at least some systemic cancers. The most obvious reason for this is the fact that many ‘regular’ biomarkers found in blood plasma are present in saliva at much lower concentrations [1], and hence those found in the former biofluid may not be readily quantifiable in the latter using this technique, although the use of more sensitive, alternative bioanalytical techniques such as LC-MS may overcome this hurdle, most especially if the analysis is targeted or semi-targeted. However, the authors are strongly encouraged by the future prospects of newly-developed NMR spectrometers with much-enhanced, operating frequency-dependent sensitivities over those currently known, which are often misunderstood or underestimated. In view of the limited information available on the sensitivity of ^1^H NMR analysis of biofluids for metabolomics assessments, we have further explored this issue and possible associated limitations in Section 4.1 of this report. 

Overall, the authors of the current study recommend that key biomarkers discovered through the use of NMR-based salivary metabolomics technologies should only be applied for the diagnosis and/or prognostic monitoring of cancer conditions when fully validated. However, full validation of such biomarkers may only be achieved once it is proven that they have the ability to respond favourably to the therapeutic application of already known or established drugs for cancer patients; unfortunately, this is certainly not the case for many published reports focused on employment of metabolomics strategies for the seeking of potential biomarkers. Moreover, it is also thoroughly recommended that when first developed, validated and accepted, metabolomics technologies should be used alongside other, more conventional and established methods for cancer diagnosis, e.g., histopathology gradings and supporting clinical and microscopic examinations, etc., in order to allow researchers to establish significant correlations between the two ‘historical’ classes of diagnoses. Once it is accepted that such correlations or associations are strong, then NMR-linked metabolomics techniques using WMSS may provide valuable diagnostic or prognostic monitoring ‘snapshots’, which may provide indispensable supporting biomolecular information; such approaches may then be employed clinically as independent diagnostic methods. 

## 10. Limitations of NMR-Based Metabolomics Investigations of Human Saliva for Cancer Diagnosis and Its Prognostic Monitoring 

In addition to problems arising from interferences with ^1^H NMR determinations of salivary APPs as described in the Section 7 Case Study above, the applications of human saliva as a medium for the detection, diagnosis and strategic monitoring of cancers is complicated by a series of issues, most especially those concerning overall experimental design (i.e., is it acceptable?), and sample collection, preparation and storage, as is evident from many metabolomics investigations conducted to date. Moreover, problems associated with the acquisition of NMR spectra, NMR pulse sequences and an acceptable understanding of their applications and purposes, along with the interpretation of spectral profiles acquired, both at higher and lower operating frequencies, should also be considered in detail prior to researchers embarking on such studies; however, these problems are covered in detail in Part 1 of this series of publications [1]. The adequate addressment of all these design and laboratory activity issues, and the enforcement of suitable precautionary measures by researchers, will serve to reinforce the validity of the experimental models applied.

Further potential disadvantages of using saliva as a diagnostic matrix for cancers include: (1) some older or vulnerable adult participants or dental patients may require quite long periods of time for the collection of samples (e.g., >20–30 min.); (2) the transfer of salivary microbes and possible biomarker agents from one human sample to another via social interactions is not impossible; (3) the presence of microbial urease, which metabolises urea to ammonia and bicarbonate via a carbamate intermediate, may serve to bias metabolomics results acquired [123]; and (4) xerostomia (dry mouth, with restricted saliva production) may occur quite frequently in some cancer patients. 

One previously reported major limitation of salivary diagnostics for cancer and other conditions, especially those of non-oral origin, has been the lower levels of putative biomarkers present when expressed relative to those in blood plasma or serum, or whole blood (often 100-fold lower or less [1]). Fortunately, with the quite recent advent and further development of bioanalytical detection platforms with enhanced sensitivities, now including that of ^1^H NMR analysis, the use of saliva as a medium for such purposes is likely to represent a future advance for the screening, identification and monitoring of cancer conditions [124]. As an example, the quantitative analysis of metabolites in WMSS samples by ^1^H NMR spectroscopy can be achieved at levels of less than only a few μmol./L in at least some cases. For example, for the metabolites specified in Section 4.1, along with Figure 3 featured therein. 

Finally, as noted in Ref. [125], a further potential limitation is problems with the use of enrichment ratios for the determination of metabolic pathway activities in order to evaluate perturbed or adversely activated biosynthetic routes in cancers and other conditions, and which employ either qualitative or quantitative metabolite set enrichment analysis (the latter abbreviated as QMSEA). Unfortunately, these strategies fail to take into account uncertainties or errors associated with assigning metabolites to specified pathways. Indeed, a hypothetical example noted in Ref. [126], which involved the attribution of three biomolecules to a single pathway with a total of eight metabolites, gave rise to a quite large enrichment ratio (3/8 = 0.375). Notwithstanding, it was noted that if all three of these pathway-associated metabolites are also active in other pathways, as indeed many are, then credence in the chemopathological involvement of that pathway from MV metabolomics datasets may be compromised, perhaps highly so. Full details of this limitation are considered further in Ref. [127].

## 11. Key Concluding Remarks and Future Perspectives 

To date, a widespread range of research effort in the metabolomics field has been focused on the development and establishment of cancer-specific biomarkers, and advantageously, many of these tend to be based on MV ‘omics’ patterns rather than single biomolecules. Indeed, as noted above in Section 8, the instigation of characteristic, perhaps metabolic pathway-linked, ‘signatures’ of say five or more biomolecules (any of which may be significantly up- or downregulated) is generally a much more reliable and accurate approach than the use of a single one. Indeed, in general, whole patterns of such dysregulated biofluid metabolite concentrations are more easily validated for clinical use than a single biomarker [128]. Nevertheless, currently only a very small number of cancer biomarkers have been adopted for routine use, and even less have been fully approved and validated for large-scale population screening or diagnosis. 

Also particularly notable is the knowledge that cancer disease symptoms are often not clearly specific until the advanced stages of the diseases explored have been reached. Therefore, the development of novel, high-throughput, rapid and reliable probes for the screening and early diagnosis of cancers is urgently required, with some attention paid to non- or virtually non-invasive technologies. To date, blood plasma has predominantly been the biofluid medium of choice for such assessments, but saliva is now becoming increasingly important as a diagnostic matrix for the seeking of cancer-linked biomarkers.

In addition to metabolites biosynthesised in the salivary glands, saliva also includes contributions from gingival crevicular fluid (GCF), serum transudate, epithelial cells, leukocytes and a very wide range of differential microorganisms. It should also be noted that GCF is an oral mucosa-generated serum transudate or inflammatory exudate, which courses into the oral cavity to contribute towards human saliva [129]. 

As documented in Ref. [1], numerous biomolecules gain entry into human saliva from blood through passive diffusion, active transport or extracellular ultrafiltration phenomena, as an essential part of the endocrine messenger portal system [130]. This passive diffusion route depends on the size and electronic charge of the molecules involved, at least in part, and this diffusion occurs from the capillaries encompassing salivary glands to acinus cells; the active transport of proteins occurs via ligand-receptor binding mechanisms; and an ultrafiltration process allowing molecules of molecular mass lower than 1900 Da to migrate through spaces located between acinus and ductal cells (transference through gap junctions between secretory elements) [131]. 

Hence, circulating biomolecules linked to the pathogenesis of diseases such as cancers, and which therefore serve as discriminatory biomarkers for them, can be transferred from the bloodstream into the salivary glands [132,133,134]. Therefore, saliva represents a diagnostic biofluid which, at least in principle, can ‘mirror’ the physiological and pathological status of the whole human body, and in principle can respond to biomolecular changes occurring in remote organ and tissue environments, despite the involvement of some likely bioanalyte concentration and sensitivity limitations. In order to confirm this, examinations of hypotheses that remote tumour-derived biomarkers, or for that matter exosomes, are mechanistically involved in the development of cancer-discriminatory salivary transcriptomic biomarkers are of critical importance for future metabolomics experiments. 

In this study, we have reported an extensive and highly up-to-date review of the employment of human saliva samples for metabolomics testing strategies designed for the diagnosis and severity monitoring of human cancer conditions, and these have included both oral and systemic diseases. These were predominantly, but not exclusively, based on the applications of high-resolution, high-field ^1^H NMR spectroscopy for multicomponent salivary analysis, and linked metabolomics strategies for determining differential, diagnostically relevant disease classifications for these samples. Of particular note, it is further stressed here that essential precautions and measures are taken to avoid the interference of exogenous agents, which unfortunately may severely confound biomarker tracking for the purpose of diagnosis and severity sub-class determinations in patients afflicted with these disorders. Although not commonly considered, one of the most common reasons for the ‘contamination’ of whole mouth saliva samples is the frequent adoption of insufficient or inappropriate fasting delay time periods prior to sample collection episodes, which are often only 1.0 h. or lower, with an average value of 1.50 h. estimated in a systematic review conducted as part of our previous study [1]. A further important precaution includes the immediate or near-immediate laboratory pre-treatment of samples with sufficient levels of a microbicidal agent in order to curtail the continued fermentation of salivary metabolites by bacteria therein during periods of sample preparation, transport and storage, and their auto-sampling analysis, by NMR or other techniques; indeed, for typical NMR analysis runs, some samples placed towards the end of autosampler runs may remain unanalysed at ambient temperature for periods of several hours or longer. An additional precautionary measure is the assurance that samples should not be stored for any longer durations than those commonly recommended in the scientific literature. 

In a bioanalytical context, all samples should also preferably be analysed as replicates in order to ensure consistency between analytical results acquired, and one reliable strategy to assess this is the performance of a PCA technique in order to check that multivariate analytical results from the same replicate samples cluster tightly together, and remain remote from those of other samples tested. 

For a single, or preferably a pathway- or other source-linked series of ‘omics’ biomarkers to be successful, their salivary concentrations should be sufficiently different and remain remote from those of corresponding normal healthy populations, preferentially during the early disease phases, and should hopefully also be useful for disease severity grading purposes. However, it should be noted that the diversified complexities of the chemopathological status of differential forms of cancers present many challenging and substantial expositions for the successful clinical employment of new ‘omics’-type biomarkers. Indeed, the broad spectrum of omics biotechnologies available still require the satisfaction of a series of technical provisions, such as bioanalytical reproducibility and precision. For example, the quite high false positive rates commonly encountered in the ‘omics’ diagnosis of cancers and other conditions serve as design barriers which have to be commonly addressed and surmounted by researchers. 

The association of determined salivary cancer biomarkers with metabolic pathways which are likely to be dysregulated in cancer cells and associated tumour biopsy tissues, and away from their normal ‘healthy’ and balanced functions, is a phenomenon of key importance in salivary metabolomics studies. However, to date, there remain investigations which do not even consider the ramifications of such pathway imbalances, and more commonly, nor do they attempt to relate or inter-relate patterns of up- or downregulated metabolites, i.e., those serving as biomarkers, for them. 

Of particular note, cancer cells consume glucose at a faster rate than their healthy counterparts, and convert glucose to lactate regardless of oxygen availability and accessibility (known as the Warburg effect) [17] via glycolysis. Moreover, a series of many cell culture experiments confirmed that glucose and amino acids, most especially glutamine for the latter, were essential requirements for cancer cell fuel purposes. Carbohydrate metabolism is of much importance since cell proliferation represents a hallmark of cancer cells, involving an enhanced level of glycolysis. Further metabolic pathways considered to be important in cancer cell homeostasis and proliferation include amino acid metabolism, notably BCAA degradation and diminished urea levels, polyamine synthesis from ornithine, lipid metabolism, oestrogen pathways, folate metabolism, nucleotide biosynthesis and last, but not least, the mitochondrial electron transport chain [29]. Indeed, the modified mitochondrial oxidative phosphorylation rate found in cancer cells is considered to be of much importance in cancer pathophysiology [134], since mitochondria serve as potent sources of ROS, which, through their aggressive chemical reactivity, exert highly salient actions regarding cancer cell apoptosis. 

Additionally, in recent years, there has been an increasing level of interest in the characterisation of glycoproteins through ^1^H-NMR analysis in order to search for reliable and robust biomarkers of diseases, including cancerous conditions. For this purpose, high-resolution ^1^H NMR analysis may at least serve to partially characterise circulating glycoproteins in WMSS samples, in addition to blood plasma or serum. However, our investigation of these analytes in the Case Study featured herein demonstrated that the ^1^H NMR resonances of at least several metabolites may also contribute to the intensity of electronically integrated APP signals in the 1.96–2.10 ppm regions of WMSS spectra acquired (specifically the GlycA one, which apparently provides diagnostic information when monitored in blood plasma). Hence, these interferants, particularly proline and glutamate, together with free N-acetylsugars or oligosaccharide species, may easily confound such measurements made on WMSS samples, as indeed does a ^13^C satellite resonance arising from the salivary-domineering acetate-CH_3_ signal. However, salivary hyaluronate was ruled out as a possible interferant in the current study. 

Overall, future prospects for the expanded use of easily collectable human saliva for the early detection of cancers, and their clinical implications and significance, are currently looking very positive. Indeed, advancements to translational and personalised medicine represent further possible options for this field. Ideally, the preliminary scientific validation of salivary metabolomics-based diagnostic markers for the identification of specific cancers should involve their correlations with clinical diagnosis criteria and histological gradings of disease severities, if indeed possible.

## Figures and Tables

**Table 2:** Scientific literature mean concentrations of biomolecules with ^1^H NMR resonances located in or close to the APP (1.96–2.10 ppm) regions of high-resolution spectra of human blood and saliva (Figure 4). ^a^ Ranges of mean values obtained from healthy control data were obtained from the *Human Metabolome Database* [101]. ^b^ Total N-acetylneuraminate level, including both APP and low-molecular-mass forms. Abbreviations: n-av, data not available.

Metabolite	Blood ^a^	Saliva ^a^
‘Free’ N-Acetylneuraminate	0.6–2.0 μmol./L	12.5–41.0 μmol./L
Total N-Acetylneuraminate ^b^	1.25–2.50 mmol./L	n-av
‘Free’ N-Acetylglucosamine	108 ± 67 nmol./L	n-av
Glutamate	24–177 μmol./L	12–14 μmol./L
Glutamine	390–905 μmol./L	5–42 μmol./L
Proline	111–259 μmol./L	6–158 μmol./L
Hydroxyproline	13–40 μmol./L	0.4–1.5 μmol./L
Lysine	105–441 μmol./L	2–59 μmol./L

## Data Availability

The data presented in this study are available in this article.
